# A bibliometric review on applications of lignocellulosic fibers in polymeric and hybrid composites: Trends and perspectives

**DOI:** 10.1016/j.heliyon.2024.e38264

**Published:** 2024-09-21

**Authors:** Caroliny M. Santos, Thiago F. Santos, H Jeevan Rao, F. Higor V.A. Silva, Sanjay Mavinkere Rangappa, Pawinee Boonyasopon, Suchart Siengchin, D.F.S. Souza, J.H.O. Nascimento

**Affiliations:** a Postgraduate Program in Chemical Engineering, Technology Center, Federal University of Rio Grande do Norte, Av. Prof. Sen. Salgado Filho, 3000, Natal, Rio Grande do Norte, 59072-970, Brazil; b Department of Mathematics, School of Sciences and Humanities, Nazarbayev University, Astana, 010000, Kazakhstan; c Natural Composites Research Group Lab, Department of Materials and Production Engineering, The Sirindhorn International Thai-German School of Engineering (TGGS), King Mongkut's University of Technology North Bangkok (KMUTNB), Bangkok, Thailand; d Department of Design Management and Business Development, Faculty of Architecture and Design, King Mongkut's University of Technology North Bangkok (KMUTNB), Bangkok, Thailand

**Keywords:** Natural fibers, Polymers, Composites, Applications

## Abstract

Over the past 10 years, materials science and engineering have shown increasing interest in incorporating lignocellulosic fibers into polymer and hybrid composites (LCF-CPH). This bibliometric analysis, covering the period 2012 to 2022, examines the current state of research on the application of these fibers in composites, with the aim of identifying significant contributions, new trends, and possible future directions. The analysis included a comprehensive database search using specific criteria, which revealed a significant increase in research activity on a variety of lignocellulosic fibers, such as flax, jute, hemp and sisal. This growth is particularly evident in the packaging, automotive, aerospace and construction industries. Hybrid composites based on these fibers have gained prominence due to their enhanced properties, which include improvements in mechanical, thermal and environmental characteristics. The findings of this research have significant implications for governments, corporations, and academic institutions. Researchers gain a deeper understanding of emerging trends, industry gains valuable insights into the advantages of adopting lignocellulosic fibers, and policymakers gain essential information to support the development of sustainable composites. In the field of advanced composites and sustainable materials, this work lays a solid foundation for future research and industrial applications.

## Highlights

• Explores the research landscape around natural fiber applications in composites.
• Examining various natural fibers for reinforcement across industries.
• Foundation for future research and industrial applications in sustainable materials.

## Graphical abstract

**Figure:**
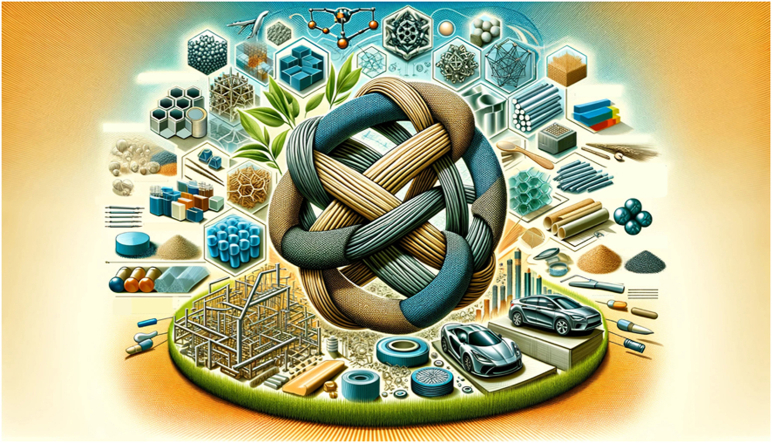

## 1 Introduction

During the last decade (2012–2022), there have been significant advances in materials, especially in lignocellulosic fiber-reinforced polymer and hybrid composites, driven by the growing awareness of sustainability and the search for environmentally friendly solutions [[1], [2], [3], [4], [5], [6]]. These developments have had a profound impact on industries such as automotive, aerospace, construction, and manufacturing [[7], [8], [9]]. Lignocellulosic fibers, such as flax, hemp, jute, sisal, and kenaf, have gained prominence as polymer reinforcements due to their comparable properties to traditional synthetic fibers like glass and carbon [[10], [11], [12], [13]]. These fibers enhance mechanical properties, including tensile strength, modulus, impact resistance, and weight reduction. Significant advancements in the treatment of fibras, formulation of matrix and fabrication of composites have further improved their mechanical properties, highlighting their potential in many applications [[14], [15], [16], [17]]. The incorporation of lignocelulosic fibres (LCF) into composites necessitates advancements in processing techniques to preserve the integrity of the fibre throughout manufacturing.

Techniques such as resin transfer molding, compression molding, and extrusion have been adapted to accommodate the unique characteristics of these fibers. Innovations in fiber extraction methods, such as enzymatic treatments and steam explosion, have enhanced fiber compatibility with polymer matrices, enabling efficient composite processing and the production of complex structures [[18], [19], [20]]. Lignocellulosic fibers, derived from renewable plant biomass, offer a vast raw material base, including fibers like kenaf, hemp, jute, and flax [[21], [22], [23]]. Their stable supply chain, driven by agricultural production, provides a competitive advantage over synthetic fibers, contributing to lower costs and reduced transportation emissions [24]. The economic viability of these fibers is further boosted by their lower production costs and the growing market demand for sustainable products, which in turn fosters job creation and economic diversification, especially in rural areas [25]. The use of LCF in composites significantly reduces environmental impact compared to traditional fibers like glass and carbon [[26], [27], [28]]. With lower CO_2_ emissions during production and the use of renewable resources, these materials support a circular economy approach [29]. Regulatory initiatives and market demand for sustainable materials have driven the growth of the natural fiber composite market, positively influencing job creation and rural economies [[30], [31], [32], [33], [34], [35]]. The use of LCF supports socio-economic development in rural communities where these materials are cultivated, aligning with Environmental, Social and Governance (ESG) principles and the UN Sustainable Development Goals (SDGs) [36]. Cultivating these fibers not only stimulates economic activity but also contributes to carbon sequestration, further enhancing their positive environmental impact. Governance aspects of ESG have become crucial in ensuring that the lifecycle of lignocellulosic fiber composites aligns with ethical and sustainability standards. Policies, regulations, and certifications guide material sourcing, production processes, and end-of-life disposal, promoting responsible consumption and production. The rise of ESG-focused funds reflects the growing interest of investors in sustainable materials and practices [37]. Thus, the integration of LCF into composite materials represents a convergence of technical innovation, economic opportunity, and environmental responsibility, driving a paradigm shift toward sustainable materials and processes.

This article offers a comprehensive analysis of LCF-PHC research from 2012 to 2022, focusing on key trends and developments in the field. Using quantitative bibliometric analysis, the study explores patterns in LCF-PHC research publications (RQ I) and identifies significant topic clusters through advanced techniques like topic cluster salience indicators and knowledge map analysis (RQ II). The goal is to provide a detailed overview of the research landscape, intellectual trajectory, and key areas of interest within the field. The article begins with Section 1 - Introduction, which outlines the theme and objectives, particularly the potential of lignocellulosic fibers in composite materials. Section 2 - Methodology and Bibliometric Approach then describes the comprehensive bibliometric analysis used to map the research landscape on lignocellulosic fibers. Section 3 - Lignocellulosic Fibers explains the fundamental characteristics of these fibers and the treatments applied to enhance their adhesion in composite matrices. Building on this, Section 4 - Fiber Surface Treatment for Good Bonding details specific surface treatments that improve bonding between the fibers and polymer matrices, crucial for the durability and effectiveness of the composites. The study continues with Section 5 - Characteristics of Publications, analyzing trends, volume, and impact of research in this field. Section 6 - Composite Manufacture discusses the methods used to manufacture composites incorporating lignocellulosic fibers, focusing on processes that ensure optimal fiber integration. Section 7 - Performance evaluates the mechanical, thermal, and environmental properties of the manufactured composites, followed by Section 8 - Sustainable Composites, which emphasizes the sustainability aspects, including their role in reducing environmental impact and supporting circular economy practices. Section 9 - Applications explores the industrial uses of lignocellulosic composites, particularly in the automotive, aerospace, construction, and packaging sectors. Finally, Section 10 - Conclusions and Future Outlook summarizes the research findings and suggests future directions and potential advancements in the field of lignocellulosic fiber composites.

## 2 Methodology

### 2.1 Bibliometric Approach

**Fig. 1:**
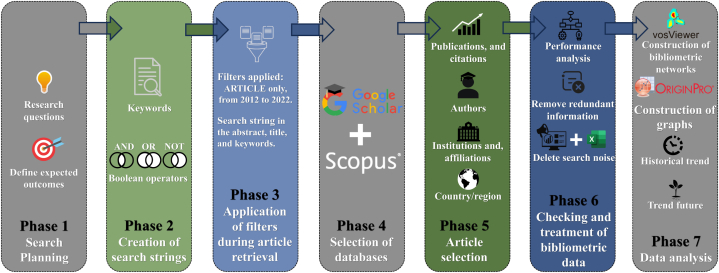
Flowchart illustrating the step-by-step process of bibliometric analysis, from data collection to data analysis.

Bibliometric analyses are increasingly utilized across various research domains, such as geopolymer composites [37], metal matrix composites [38], the recycling of fiber-reinforced plastics [39], thermal energy storage in cement composites with phase change materials [40], rice straw/husk polymer composites [41], and magnesium oxychloride cement-based building materials [42]. This analytical approach has become particularly prominent in composite materials research, providing critical insights and trends. Recent noteworthy bibliometric studies in this field have been conducted by researchers such as Yang H. in 2022 [37], Sekhar R. in 2021 [38], Colombo B. in 2022 [39], Afgan S. in 2021 [40], Deepaky D. in 2021 [41], and Maier A. in 2022 [42]. These studies employ sophisticated bibliometric tools, like co-citation and social network analysis, to map the landscape of research progression, highlight emerging topics, and analyze the impact of publications within the field. The bibliometric methodology applied to evaluate the use of LCF-PHC from 2012 to 2022 was based on the frameworks established by Borges P.T. et al., in 2023 [43], Lee M.B. et al., in 2023 [44], and Moutik B. et al., in 2023 [45]. This involved a meticulous process that included selecting data sources, defining search strategies, setting criteria for what studies to include or exclude, and choosing the appropriate data analysis methods as shown in Fig. 1.

### 2.2 Data sources

Comprehensive academic databases Scopus, and Google Scholar were utilized to retrieve relevant scholarly articles and publications. Citation Databases: Citation databases like Google Scholar were also accessed to collect information on citations and citation networks. The search strategy employed a combination of relevant keywords and Boolean operators to ensure a comprehensive and focused retrieval of articles. The primary search string used was: ("natural fibers AND hybrid OR composites OR polymeric AND composites AND mechanical OR tensile, OR chemical, OR thermal OR morphological OR properties OR packaging, OR conventional, OR aerospace, OR automotive, OR sporting, OR marine OR oil AND industry, OR building, OR applications"). This search string aimed to capture articles that encompassed various aspects of LCF in composites, including mechanical, chemical, thermal, and morphological properties, as well as applications in different industries and sectors.

### 2.3 Inclusion and exclusion criteria

To ensure the relevance and quality of the retrieved articles, the following inclusion (articles published between 2012 and 2022, in English, related to the applications of LCF-PHC. The exclusion criteria were applied (articles published before 2012 or after 2022, in languages other than English, unrelated to the study's focus on LCF in composites.

### 2.4 Data collection

The data collection process involved systematically searching selected databases using defined criteria. Article metadata, such as title, abstract, and keywords, were extracted. Keywords were analyzed to identify common themes and trends. Citation networks were constructed to identify influential elements in the domain. Publication trends over time were examined to spot growth patterns and research hotspots. Data analysis focused primarily on article titles, abstracts, and keywords, providing a comprehensive view of the research landscape from 2012 to 2022. Through systematic search, clear criteria, and various analysis techniques, the bibliometric analysis aimed to offer a robust examination of LCF-PHC research during the specified period.

## 3 Lignocellulosic fibers

### 3.1 Tropical zones

In the tropical zones where climatic conditions permit year-round cultivation, a variety of LCF have been produced continuously over the past decade (2012–2022), each finding unique applications in the field of polymeric and hybrid composites. Kenaf (Hibiscus cannabinus) and Elaeis guineensis (oil palm derived fibers-OPEFB) [[46], [47], [48]] are plants of great value due to its exclusive properties, such as resistance, durability and lightness [49,50]. Its fibers are highly versatile and find application in various sectors, such as paper, textiles, construction and biocomposites [23,51,52]. Cissus quadrangularis [53] and Epipremnum aureum [54] have been explored for their potential in biocomposites due to their robust growth and ease of extraction. Boehmeria Nivea (Ramie) [55,56] is particularly noted for their high tensile strength and have been used in automotive and construction materials. Musa textilis (Abaca), known for its high mechanical strength relative to other natural fibers, has been used in the manufacture of specialty papers, ropes, and even automotive composites for interior applications [57]. Dichrostachys Cinerea [58,59], not as widely known, offers prospects for material reinforcement, similarly to Acacia Leucophloea [60,61] and Trachelospermum jasminoides [62], which are being investigated for their fibrous properties. Sida Cordifolia [63] and Carica Papaya [64,65] have seen experimental applications in composite materials due to their abundance and renewability. Jute (Corchorus olitorius) is recognized as one of the most widely accepted and produced natural fibers globally. Thanks to its affordable cost and high specific strength, it has been extensively used in composites for packaging, furniture and the automotive industry [66,67]. Thespesia Populnea [68,69], Agave sisalana (Sisal) [70], and Fimbristylis globulosa [71,72] fibers have been incorporated into composites used in furniture, geotextiles, and as alternatives to glass fibers in reinforced plastics due to their high durability and low density. Coccinia grandis [73,74] and Mucuna atropurpurea [75], while less common, have seen niche applications in composites. Tridax procumbens [76] has been studied for its potential use in paper and packaging composites. The versatility and continuous availability of these fibers have encouraged their exploration in various sectors, with researchers focusing on their biodegradability, mechanical properties, and sustainability profiles. The last decade has underscored the importance of these fibers in driving economic growth within the tropical regions where they are produced. Their integration into the composite material market reflects a sustainable approach to industrial development, leveraging renewable resources that are less energy-intensive and more environmentally benign compared to traditional synthetic fibers. This shift towards LCF in composites is aligned with global sustainability goals and is likely to continue as both technology and environmental regulations evolve.

### 3.2 Temperate zones

In temperate zones, the production of LCF is typically constrained by the climatic conditions, with harvests generally limited to the summer and autumn seasons. This limitation imposes a distinct rhythm on the supply chain of fibers such as Hierochloe Odarata [77], Cereus Hildmannianus [78], Conium maculatum [79,80], and fibers from the Red banana peduncle [81]. Despite these seasonal restrictions, these fibers have found specialized applications in the field of PHC over the last decade. Hierochloe Odarata, known for its sweet scent and strength, has been utilized in traditional handcrafting and has seen modern applications in the development of niche composites for interior design and eco-friendly products. Due to its limited seasonal availability, its use is often reserved for small-scale, high-value items. Cereus Hildmannianus, a cactus native to South America but also grown in temperate regions, yields fibers that have been studied for their utility in reinforcement applications within composite materials [78,82]. These fibers are particularly interesting due to their resilience and potential for use in non-structural composites [83]. Conium maculatum, despite its toxicity, has been researched for its fibrous content. However, due to the health risks associated with its handling, its application has been largely experimental and not widely commercialized [80]. Fibers from the Red banana peduncle have been explored for their potential in textile and composite applications, contributing to the development of environmentally friendly materials. Flax (Linum usitatissimum) is the most widely used natural fibers globally, due to their low cost and high specific strength. Its application spans diverse industries, including packaging, furniture and automotive [67,84]. Cannabis sativa (Hemp) [79,80] is particularly noted for their high tensile strength and have been used in automotive and construction materials. Taken together, flax, hemp and nettle are examples of LCF common in the temperate zone, which play an important role in the production of composites and benefits for a more sustainable and ecologically conscious economy.

### 3.3 Reinforcement

Over the decade spanning 2012 to 2022, the utilization of sustainable natural fibers, derived from agricultural and recycled sources (LCF), has surged, supplanting synthetic fibers and bolstering environmental conservation endeavors. This shift towards sustainability has been instrumental in shaping preferences, particularly in the realm of composite materials. Notably, in the reinforcement of Polymer Hybrid Composites (PHC), there has been a marked evolution with a diversified array of reinforcement types.

**Fig. 2:**
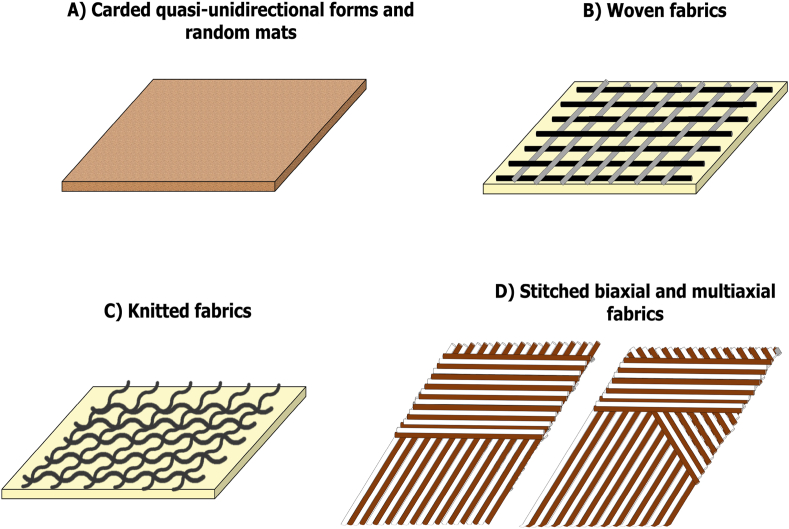
Classification of reinforcements for lignocellulosic fiber-reinforced polymeric and hybrid composites.

Carded quasi-unidirectional forms and random mats serve distinct purposes, catering to applications necessitating specific directional strengths or isotropic properties, respectively as showed in Fig. 2A [85,86]. Quasi-unidirectional reinforcements, prized for their high longitudinal strength, are prominently employed in automotive panels [87]. On the other hand, random mats find their niche in interior automotive components and construction materials due to their multidirectional strength and molding ease [88]. Woven fabrics, characterized by two sets of yarns or fibers interlaced perpendicularly, are renowned for their high tensile strength and dimensional stability as showed in Fig. 2B [89]. Their versatility makes them indispensable in aerospace, automotive, marine, and sporting goods sectors, offering substantial strength and stiffness with minimal weight [90]. Among various weave types, plain weave stands out for its simplicity and balanced properties, making it a popular choice [91]. Knitted fabrics, formed by interlocking yarn loops, offer a more flexible and stretchable alternative to woven fabrics as showed in Fig. 2C [92,93]. This flexibility renders knitted reinforcements ideal for applications demanding deformability and conformability, such as in complex-shaped automotive and aerospace components [94,95]. Knitted reinforcements are available in weft and warp knit varieties, with weft knits being favored for their simpler manufacturing process and superior stretchability. Stitched reinforcements entail sewing or stitching yarns through fabric layers, imparting a third dimension to the material structure [96]. This technique enhances out-of-plane properties like impact resistance and delamination strength, making stitched fabrics invaluable in military, defense, sporting equipment, and marine vessel construction [96,97]. Notably, stitched biaxial and multiaxial fabrics, offering tailored directional strength and rigidity, have witnessed increased adoption in composite materials from 2012 to 2020 as showed in Fig. 2D. This trend is propelled by the quest for lightweight, high-strength, and sustainable solutions across diverse industries [98]. Innovations in fiber surface treatments and composite manufacturing techniques have further bolstered the compatibility and performance of these reinforcements, driving the advancement of composite materials.

## 4 Fiber surface treatment for good bonding

### 4.1 Alkaline treatment

The period from 2012 to 2022 has seen significant advancements in the field of lignocellulosic fiber-reinforced composites, particularly in the area of fiber surface treatments to enhance material properties. Among these, alkaline treatment, also known as mercerization, has been a focal point of research due to its effectiveness in improving the interfacial adhesion between fibers and polymer matrices. Alkaline treatment involves the immersion of LCF in a sodium hydroxide (NaOH) solution. This process works by removing natural and surface impurities such as waxes, oils, and lignin, which cover the cellulose microfibrils. The primary mechanism is the saponification of intermolecular ester bonds in lignin and hemicellulose, leading to a more exposed and rougher fiber surface. This increase in surface roughness enhances the mechanical interlocking and chemical bonding capabilities between the fiber and matrix materials. The effectiveness of alkaline treatment depends on several parameters, including the concentration of NaOH solution (typically ranging from 1 % to 10 %) [[99], [100], [101]], the treatment duration (from 10 min to 12 h) [[101], [102], [103]], the temperature of the solution [102], and the fiber type [100,103]. Studies have consistently shown that alkaline-treated fibers contribute to composites with higher tensile strength, flexural strength, and impact resistance compared to untreated fiber composites [[104], [105], [106]]. The removal of lignin and hemicellulose results in increased surface roughness and the exposure of more hydroxyl groups on cellulose, improving the chemical bonding potential with polymer matrices [107]. By removing hydrophilic components and increasing compatibility with hydrophobic matrices, alkaline treatment helps reduce the moisture absorption of the composites, which is crucial for maintaining mechanical properties in humid conditions [108].

### 4.2 Silane treatment

Over the last decade (2012–2022), the surface modification of LCF using silane coupling agents has garnered significant attention in the realm of polymer composite research [[109], [110], [111]]. Silanes, characterized by their general formula SinH_2_n+2, such as silicon alkoxides, possess a unique dual nature, hydrophilic properties coupled with various functional groups linked to the silicon atom. This duality enables silane molecules to serve as effective bridging agents within composite materials, facilitating enhanced interaction between hydrophilic LCF and the hydrophobic polymer matrix. The mechanism of silane treatment involves multiple stages, each influenced by specific parameters like heating, pH levels, hydrolysis reaction time, and the functionality of the silane used. Initially, silane monomers undergo hydrolysis in the presence of water and a catalyst (either an acid or a base), leading to the formation of reactive silanol groups while releasing alcohol [112]. This step is crucial for preparing the silane for bonding with the fiber surface. To ensure effective bonding, the process aims to minimize self-condensation during hydrolysis, preserving silanol groups for subsequent interaction with the cellulose's hydroxyl groups within the fibers. The physical adsorption of silanol groups onto the fiber surface or within the cell walls is primarily facilitated through hydrogen bonding, creating a surface coating or causing swelling in the cell wall. Furthermore, these reactive groups can undergo self-condensation to form a stable polysiloxane network, characterized by durable -*Si*-O-Si- bonds, enhancing the composite's structural integrity. The application of heat plays a pivotal role in transforming these initial interactions into stronger bonds. At elevated temperatures, hydrogen bonds between silanol and cellulose hydroxyl groups evolve into covalent bonds (-Si–OC–), with water being a by-product of this condensation reaction [113]. This stage, known as grafting, is critical for achieving robust and durable bonding between the LCF and the matrix. This covalent bonding mechanism not only strengthens the interface but also significantly enhances the mechanical properties and durability of the composite material [114].

### 4.3 Acetylation treatment

Emerged as a pivotal technique for enhancing the compatibility and bonding between LCF and polymer matrices from 2012 to 2022. This chemical modification method aims to alter the surface characteristics of natural fibers, reducing their inherent hydrophilicity and augmenting their thermal stability [115,116]. The core principle of acetylation involves the substitution of the fiber's hydroxyl groups (-OH) with acetyl groups (CH3CO-), thereby imparting a hydrophobic character to the fiber surface [117]. LCF contain various hydroxyl groups associated with cellulose, hemicelluloses, and lignin. These -OH groups are responsible for the fibers' hydrophilic nature, leading to moisture absorption that adversely affect composite material properties [118]. Acetylation directly addresses this challenge by converting these hydrophilic groups into hydrophobic acetyl groups. The process predominantly targets the more reactive hydroxyl groups in hemicelluloses and lignin, as cellulose itself is less reactive due to its crystalline structure. Given cellulose's resistance to direct acetylation, acetic anhydride is favored over acetic acid for its efficiency in facilitating this chemical transformation. The acetylation process not only enhances the thermal stability of LCF but significantly improves their compatibility with polymer matrices [119]. By making the fiber surface hydrophobic, acetylation reduces the risk of moisture-induced degradation within the composite material. This reduction in moisture absorption minimizes the potential for swelling and shrinkage, which lead to weak points or failures at the fiber-matrix interface. Furthermore, the modified surface improves mechanical interlocking and chemical bonding possibilities with various matrix materials, leading to composites with superior mechanical properties and durability [120]. These advancements have led to the development of natural fiber-reinforced composites with enhanced performance in automotive, construction, and packaging applications, where moisture resistance and mechanical integrity are paramount.

### 4.4 Enzymatic treatment

The employment of bio-catalysts, specifically enzymes produced by microorganisms, has emerged as a favored approach for the treatment of lignocellulosic fibers, spotlighting its beneficial environmental footprint. Enzymatic agents like amylase, cellulase, protease, and catalase are increasingly utilized to expedite chemical reactions under mild conditions-often at low concentrations and temperatures nearing ambient levels—facilitating the biodegradation of cellulose. This method stands out for its eco-friendly nature, circumventing the need for harsh chemicals and high-energy processes typically associated with lignocellulosic fiber treatment. A notable study by Bledzki on polypropylene (PP) composites reinforced with abaca fibers subjected to enzymatic treatment underscores the efficacy of this approach [121,122]. The investigation revealed that enzymatic processing could proficiently remove natural binders, such as waxes and lignin that commonly coat the surface of raw fibers. This purification process is critical for enhancing the interfacial adhesion between the fibers and the polymer matrix, a key factor in the mechanical performance of the composites [121]. However, the study also brought to light a potential drawback of enzymatic treatment in fibrillation and potential damage to the fibers. While the process excels in removing unwanted surface components, it can also lead to the weakening of fiber integrity if not carefully controlled. This highlights the importance of optimizing treatment conditions to balance the benefits of surface cleaning with the preservation of fiber strength. The exploration of enzymatic treatments for LCF points toward a sustainable pathway for composite material production, aligning with broader environmental objectives. Future research in this area is likely to focus on refining the application of enzymes to minimize fiber damage while maximizing the environmental and performance advantages of such treatments. This approach not only contributes to the development of more sustainable composite materials but also opens up new possibilities for utilizing LCF in a variety of applications.

## 5 Characteristics of publications

**Fig. 3:**
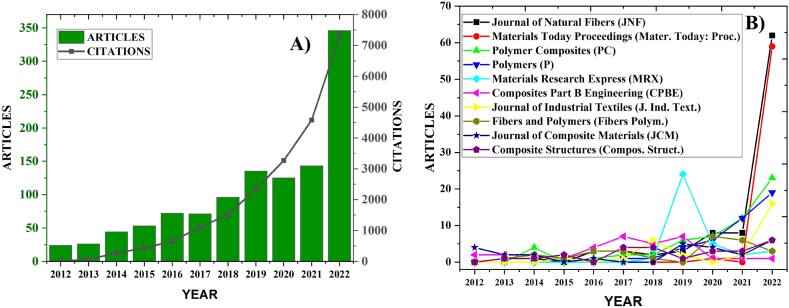
Articles published annually from 2012 to 2022 in LCF-PHC.

Fig. 3A and B, illustrate the scholarly activity in the area of LCF-PHC over the last decade. Fig. 3A demonstrated a clear trend of growth in both the number of articles published and the number of citations received over the years 2012–2022. The number of articles started at a relatively low point in 2012 and has shown a steep increase, peaking in 2022. Similarly, citations have followed an upward trajectory, which suggests that the research produced is gaining recognition and is considered valuable by the scientific community. The significant rise in citations indicate that the work is foundational or highly relevant to ongoing research in the field. Fig. 3B presents a breakdown of articles published by various journals in the same field. The top 10 journals, based on the number of articles published in 2012–2022, were Journal of Natural Fibers (JNF), Materials Today Proceedings (Mater. Today: Proc.), Polymer Composites (PC), Polymers (P), Materials Research Express (MRX), Composites Part B Engineering (CPBE), Journal of Industrial Textiles (J. Ind. Text.), Fibers and Polymers (Fibers Polym.), Journal of Composite Materials (JCM), and Composite Structures (Compos. Struct.). In the last decade, research into lignocellulosic fiber composites has surged, reflected by increased publications in leading journals. These journals (JNF, Mater. Today: Proc., PC, P, MRX, CPBE, J. Ind. Text., Fibers Polym., JCM, and Compos. Struct.) have been instrumental in sharing knowledge on lignocellulosic fiber extraction, processing, and application, serving as a primary outlet for new findings in this field. They've captured cutting-edge research from conferences, explored the integration of fibers into polymer matrices, and covered the full spectrum of scientific inquiry from fiber-polymer interactions to practical engineering applications. Additionally, they've addressed industrial and engineering applications, bridging theoretical and practical aspects of lignocellulosic fiber composites. These contributions have been crucial for the advancement of sustainable composite materials and their industrial applications.

**Fig. 4:**
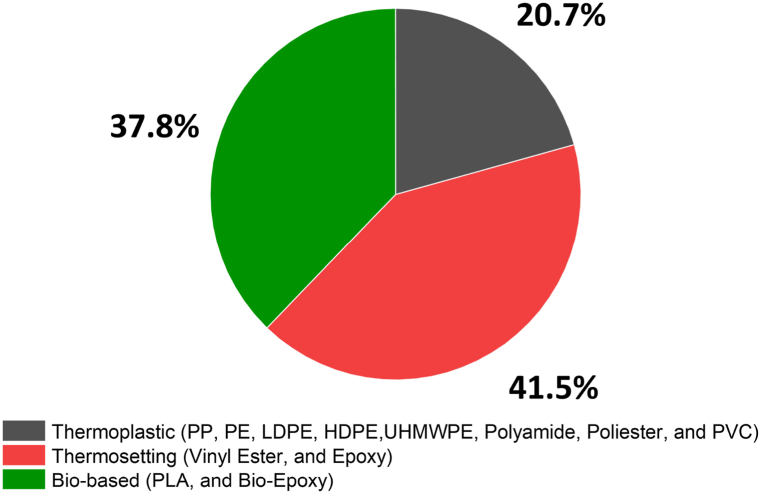
Matrix systems used in LCF-PHC from 2012 to 2022.

Fig. 4 illustrates the proportional use of different matrix systems for LCF over a decade, from 2012 to 2022. Fig. 4 is divided into three major segments, representing thermoplastic, thermosetting, and bio-based systems. From Fig. 4, was exhibited that thermoplastic matrix systems account for the largest share, with 41.5 %. This category includes a variety of polymers such as polypropylene (PP), polyethylene (PE), low-density polyethylene (LDPE), high-density polyethylene (HDPE), ultra-high molecular weight polyethylene (UHMWPE), polyamide, polyester, and polyvinyl chloride (PVC). Among these, polyester has been the most utilized matrix in thermoplastic systems for lignocellulosic fibers. Thermosetting systems comprise the second-largest category, with 37.8 % of the total. This includes vinyl ester and epoxy matrices. Notably, the epoxy matrix has been the most frequently used among thermosetting systems in this context, which is often due to its excellent mechanical properties and adhesive characteristics, making it a preferred choice for composite materials. Lastly, the bio-based systems, which include polylactic acid (PLA) and bio-epoxy, make up 20.7 % of the matrix systems used. Within this category, PLA has been the predominant matrix used with lignocellulosic fibers. PLA is known for its biodegradability and is derived from renewable resources, which adds to its appeal in an increasingly environmentally conscious market. Thus, the data highlights a significant inclination towards thermoplastic systems, with a strong preference for polyester matrices within this category. The prominence of epoxy matrices in thermosetting systems underscores their critical role in composite materials. Moreover, the substantial portion of bio-based systems, led by PLA, indicates a growing trend towards sustainable materials in the industry. This shift could be reflective of the rising demand for green composites and the industry's response to environmental concerns.

### 5.1 The most cited authors

**Fig. 5:**
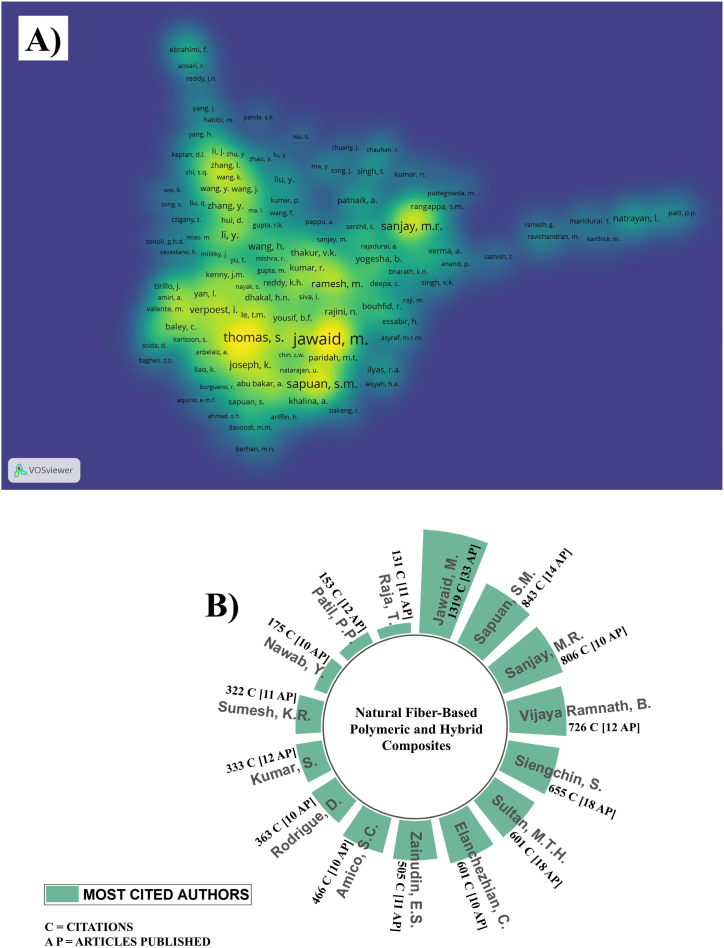
A) Co-citation density is represented through a color-coded system, where each data point, corresponding to an author, is assigned a color that signifies the concentration of citations per author. The spectrum of colors acts as a density indicator, with yellow marking a higher density of cited articles for a given author and blue representing a lower citation density. B) Visualization of highly cited authors in LCF-PHC (2012–2022).

In Fig. 5, was recognize and celebrate between 2012 and 2022 the top 15 most prominent researchers such as Dr. Jawaid, M.; Dr. Sapuan, S.M.; Dr. Sanjay, M.R.; Dr. Vijaya Ramnath, B.; Dr. Siengchin, S.; Dr. Sultan, M.T.H.; Dr. Elanchezhian, C.; Dr. Zainudin, E.S.; Dr. Amico, S.C.; Dr. Rodrigue, D.; Dr. Kumar, S.; Dr. Sumesh, K.R.; Dr. Nawab, Y.; Dr. Patil, P.P.; and Dr. Raja, T.; have consistently produced high-impact research in the realm of lignocellulosic fiber composites as shown in Fig. 5A. These experts have made pivotal contributions to scientific understanding and industrial applications, significantly influencing the field. Dr. Jawaid, M., leading the list, is renowned for his extensive research at Universiti Putra Malaysia, with over 600 publications and 34,973 citations. His H-index exceeds 90, underlining his global academic impact. Following him, Dr. Sapuan, S.M., also from Universiti Putra Malaysia, boasts over 1000 publications, 34,048 citations, and an H-index above 95. In third place, Dr. Sanjay, M.R., from King Mongkut's University of Technology North Bangkok, has 17,010 citations and an H-index above 67, reflecting his significant contributions in Mechanical Engineering and Lignocellulosic Fiber Composites. Other noted researchers include Dr. Vijaya Ramnath, B., Dr. Siengchin, S., Dr. Sultan, M.T.H., Dr. Elanchezhian, C., Dr. Zainudin, E.S., Dr. Amico, S.C., Dr. Rodrigue, D., Dr. Kumar, S., Dr. Sumesh, K.R., Dr. Nawab, Y., Dr. Patil, P.P., and Dr. Raja, T. They are recognized for their extensive contributions to composites, with notable citation counts and H-indices. Each has authored a significant number of research papers and articles, making profound impacts in their respective fields. Fig. 5B collectively showcases the researchers' academic contributions, promoting the field through their extensive research on lignocellulosic fiber composites. Their work, as reflected by the number of citations and articles published, has likely advanced scientific understanding and influenced industrial applications of these materials. This serves as a testament to the importance of their research and its role in driving innovation and knowledge in the science of composites.

### 5.2 The most productive countries/territories

**Fig. 6:**
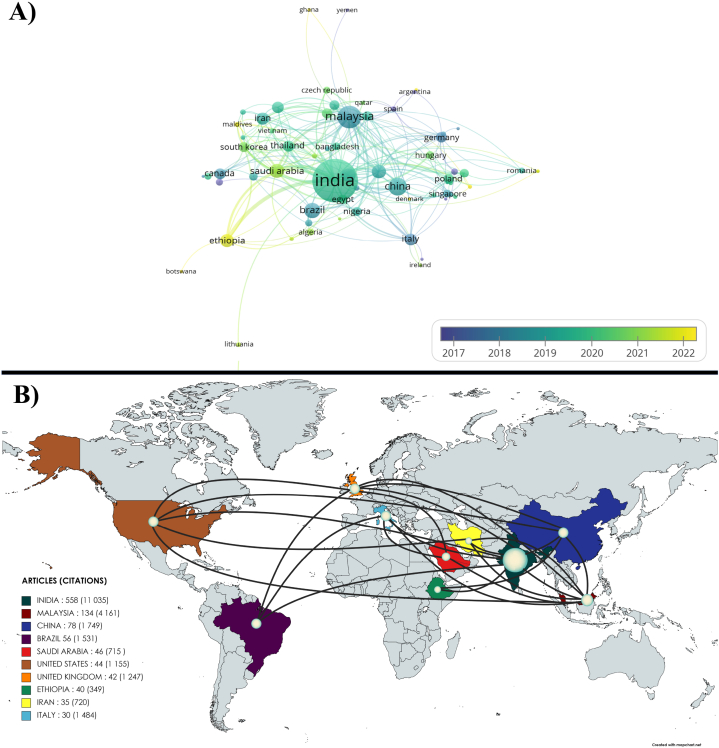
A) Overlay visualization of co-authorship countries, B) Geographical distribution of publication (2012–2022) in lignocellulosic fiber-based polymeric and hybrid composites.

Fig. 6A shown the countries have demonstrated their commitment to advancing research in lignocellulosic fiber composites, aligning with the 2030 agenda's goals for sustainability and innovation. Their concerted efforts, investments, and policy initiatives have propelled them to the forefront of this field, contributing to eco-friendly materials and innovative applications. Countries like India, Malaysia, China, Brazil, Saudi Arabia, United States, United Kingdom, Ethiopia, Iran, and Italy have been instrumental in shaping the trajectory of research in lignocellulosic fiber composites as shown in Fig. 6B. Their concerted efforts, investments, and policy initiatives have propelled them to the forefront of this field.

India, Malaysia, China, Brazil, Saudi Arabia, the United States, the United Kingdom, Ethiopia, Iran, and Italy are among the top countries leading the charge in high-impact research on lignocellulosic fiber composites, a field critical for sustainable development. India tops the list with 558 articles, an h-index of 154, and 11,035 citations, boasting an average citation rate of 19.8 Citations/TP. Malaysia follows with 134 articles, an h-index of 51, and 4161 citations. China's commitment is reflected in its 78 articles, 1749 citations, and an h-index of 24. Brazil has published 56 articles, received 1531 citations and held an h-index of 18. Saudi Arabia, with 46 articles and an h-index of 16, has garnered 715 citations. The United States, in 6th place, has contributed 44 articles with 1155 citations and an h-index of 14. The UK's research has led to 42 articles, 1247 citations, and an h-index of 17. Ethiopia's growing research presence is indicated by its 40 articles, 349 citations, and an h-index of 14. Iran, with an h-index of 16, has 720 citations across 35 articles. Italy rounds out the top ten with 30 articles, 1484 citations, and an h-index of 13. These countries' significant research outputs underscore their vital role in advancing polymeric composite science. Their concerted efforts in pushing the boundaries of materials science are aligned with the UN's Sustainable Development Goals, particularly those on responsible consumption, production, and climate action. These countries collectively enhance the polymeric composite science field through investments, policy initiatives, and sustainable practices. The values of articles published, h-index, citations, and Citations/TP highlight the tangible impact of their contributions to the field as shown in Table 1.

**Table 1:** Top ten of the country publications and citations for “lignocellulosic fibers; hybrid composites; polymeric composites”.

S. No	Country	TP (R%)	h-index	Citations (Rank)	Citations/TP (Rank)
1	India	558 (49.2 %)	154	11,035 (1°)	19.8 (8°)
2	Malaysia	134 (11.8 %)	51	4161 (2°)	31.1 (2°)
3	China	78 (6.9 %)	24	1749 (3°)	22.4 (6°)
4	Brazil	56 (4.9 %)	18	1531 (4°)	27.3 (3°)
5	Saudi Arabia	46 (4.1 %)	16	715 (9°)	15.5 (9°)
6	United States	44 (3.9 %)	14	1155 (7°)	26,3 (5°)
7	United Kingdom	42 (3.7 %)	17	1247 (6°)	26.7 (4°)
8	Ethiopia	40 (3.5 %)	14	349 (10°)	8.7 (10°)
9	Iran	35 (3.1 %)	16	720 (8°)	20.6 (7°)
10	Italy	30 (2.6 %)	13	1484 (5°)	49,5 (1°)

**TP:** Total journal publications; **R%:** Rank.

### 5.3 The most productive institutions

**Fig. 7:**
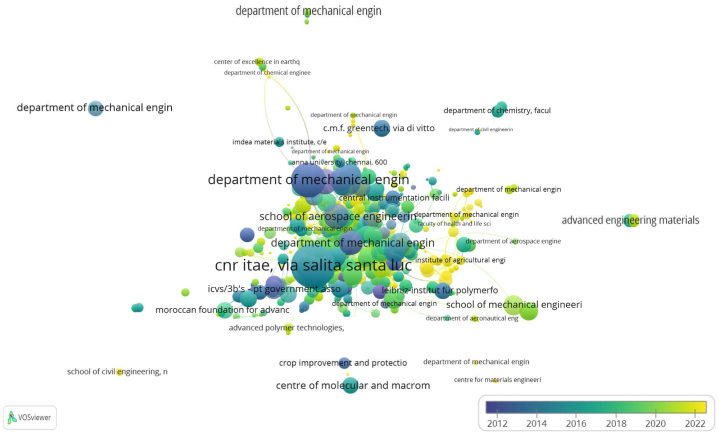
Overlay visualization represents the cumulative number of citations of articles focused on LCF-PHC across various institutional departments over a period from 2012 to 2022. The color of each term within the visualization indicates the average citation timeline by department, whereas the dimension of the nodes correlates with the total frequency of citations.

The provided Table 2 and Fig. 7 together offer a comprehensive overview of the impact and distribution of research in the field of LCF-PHC from 2012 to 2022. Table 2 lists the top ten institutional affiliations that have garnered the most citations (TC) for research in this area, highlighting the influence and contribution of these institutions within the scientific community. Table 2 also includes a percentage weightage, which provides insight into the relative impact each institution has in terms of citations compared to the total. Additionally, the countries of these institutions are noted, indicating the geographic distribution of research leadership in this domain. CNR ITAE, Italy exhibited a total citation count of 867 and a percentage weightage of 17.9 %, this institution tops the list, indicating a significant impact on the research community. Department of Ingegneria Civile, Italy, matching CNR ITAE with the same total citations and percentage weightage, this department appears to be a leading contributor as well. Department of Mechanical Engineering (Anna University), India shows a considerable citation count of 535, contributing to 11.1 % of the total weightage. Department of Mechanical Engineering (Dr. Mahalingam College of Engineering and Technology), India, also with 535 TC and 11.1 % weightage, indicating a strong research presence. Department of Mechanical Engineering (Jawaharlal Nehru Technological University), India matching the fourth position in terms of citations and weightage. Department of Mechanical Engineering (Sri Sai Ram Institute of Technology), India, has a total citation count of 400, holding 8.2 % of the total weightage. Department of Mechanical Engineering (Sri Sairam Engineering College), India, exhibited 293 citations, it accounts for 6.1 % of the weightage. Jawaharlal Nehru Technological University, India another significant contributor with 283 citations and 5.8 % weightage. School of Aerospace Engineering and Applied Mechanics, China, presented 270 citations, it has a weightage of 5.5 %. School of Materials and Mineral Resources Engineering, Malaysia, rounds out the list with 258 citations and a weightage of 5.3 %.

**Table 2:** Top ten of the most relevant institutional affiliations (2012–2022) for the topic of lignocellulosic fiber-based polymeric and hybrid composites.

S. No	Institute affiliation	TC	% Weightage	Country
1	CNR ITAE	867	17.9	Italy
2	Department of Ingegneria Civile	867	17.9	Italy
3	Department of Mechanical Engineering (Anna University)	535	11.1	India
4	Department of Mechanical Engineering (Dr. Mahalingam College of Engineering and Technology)	535	11.1	India
5	Department of Mechanical Engineering (Jawaharlal Nehru Technological University)	535	11.1	India
6	Department of Mechanical Engineering (Sri Sai Ram Institute of Technology)	400	8.2	India
7	Department of Mechanical Engineering (Sri Sairam Engineering College)	293	6.1	India
8	Jawaharlal Nehru Technological University	283	5.8	India
9	School of Aerospace Engineering and Applied Mechanics	270	5.5	China
10	School of Materials and Mineral Resources Engineering	258	5.3	Malaysia

**TC:** Total citations.

Fig. 7 shown an overlay visualization that graphically represents the cumulative number of citations across different institutions. The color gradient applied to each term indicates the average citation timeline, with the color spectrum typically corresponding to a timeline from earlier years (often represented by cooler colors like blue) to more recent years (often represented by warmer colors like red). The size of each node (term) represents the total number of citations, with larger nodes indicating a higher frequency of citations. This visual representation allows for an at-a-glance understanding of which institutions have been most active and influential in the field over the specified period. The sizeable presence of Indian institutions, occupying six out of the ten spots, suggests a strong emphasis and contribution to this area of research within India. The appearance of institutions from Italy, China, and Malaysia underscores the international interest and collaborative nature of research in lignocellulosic fiber-based materials. The significant citation counts reflect the global importance of sustainable materials research, with LCF being a focal point due to their renewable nature and potential for reducing reliance on non-renewable resources.

### 5.4 The most researched areas

**Fig. 8:**
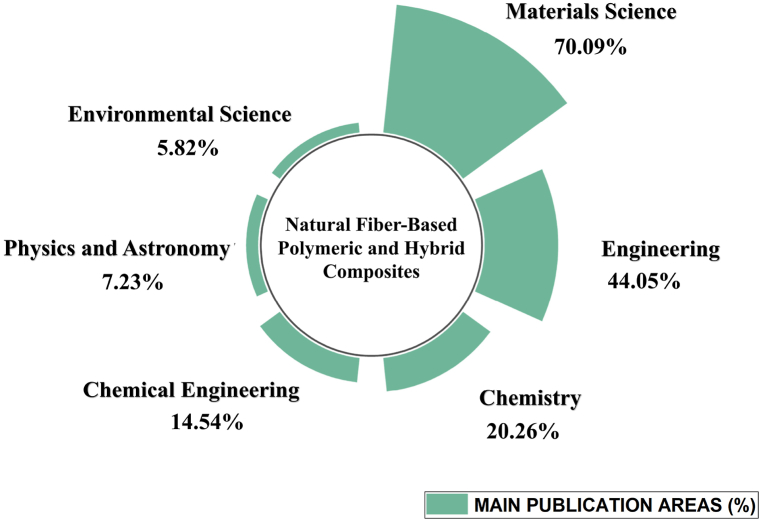
Top 5 Areas of Publications (2012–2022) for lignocellulosic fiber-based polymeric and hybrid composites.

The distribution of publications across various research areas, including Materials Science, Engineering, Chemistry, Chemical Engineering, Physics and Astronomy, and Environmental Science, in the field of LCF-PHC from 2012 to 2022 was attributed due to LCF-PHC are inherently interdisciplinary materials as shown in Fig. 8 [123]. They involve aspects of materials science, engineering, chemistry, and chemical engineering in their development, characterization, and application. This interdisciplinary nature naturally leads to publications in multiple fields. Materials Science (70.09 %) was leading area of publication because it serves as the core discipline for studying and developing composite materials. Researchers in Materials Science explore aspects such as fiber properties, matrix materials, manufacturing processes, and material characterization. Engineering (44.05 %) closely follows Materials Science due to its critical role in the practical application of composites. Researchers in Engineering often focus on designing and optimizing composite structures for specific applications, such as automotive components or structural elements. Chemistry (20.26 %) is essential for understanding the chemical interactions between LCF and polymer matrices. This knowledge is crucial for tailoring composite properties and improving compatibility. Chemical Engineering (14.54 %) contributes to the development of composite manufacturing processes and the scale-up of production. It also addresses issues related to processing and quality control. Physics and Astronomy (7.23 %): Physics and Astronomy may play a role in studying the physical properties of composites, such as their mechanical behavior and thermal properties. Environmental Science (5.82 %) contributes positively to the lignocellulosic fiber-based composites, because they are often considered more environmentally friendly than traditional composites, which aligns with the interests of researchers in Environmental Science. They investigate the sustainability and environmental impact of these materials. Based on the data of bibliometric analysis, the top 5 areas of publications in the field of LCF-PHC from 2012 to 2022 are Materials Science, Engineering, Chemistry, Chemical Engineering, Physics and Astronomy as shown in Fig. 8. These areas reflect the multidisciplinary nature of research in this field, with Materials Science and Engineering leading the way due to their fundamental roles in the development and application of these composite materials. Chemistry and Chemical Engineering are also prominent because they address the intricate chemical and processing aspects. Physics and Astronomy play a supporting role in understanding the physical properties of the composites.

### 5.5 Analysis by journal sources

In the dynamic field of lignocellulosic fiber composite research spanning the years 2012–2022, several journals have consistently stood out for their significant contributions, impact, and relevance as shown in Table 3. The Journal of Natural Fibers ranks first, with 91 articles and an h-index of 47, supporting sustainable practices in line with the UN's 2030 agenda. Materials Today: Proceedings follows, with 60 articles and an h-index of 69, showcasing sustainable material research and contributing to UN Sustainable Development Goals (SDGs). Polymer Composites is third with 57 articles, an h-index of 94, and a CiteScore of 6.7, reflecting its role in sustainable material development. Polymers, in fourth place, has published 43 articles and boasts an h-index of 113 and a CiteScore of 7.2, disseminating influential research in polymer science. Materials Research Express, with 35 articles and an h-index of 52, is fifth, advancing materials science research. Composites Part B: Engineering, in sixth place, features 32 articles with a high h-index of 184 and a leading CiteScore of 23.2, highlighting its significant impact. Seventh-ranked Journal of Industrial Textiles has published 28 articles and holds an h-index of 49, contributing to the development of industrial applications of lignocellulosic fibers. Fibers and Polymers, with 27 articles and an h-index of 65, stands eighth, supporting advancements in fiber science. The Journal of Composite Materials, ninth, with 26 articles and an h-index of 102, promotes the study of composite materials including lignocellulosic fibers. Finally, Composite Structures, in tenth place, has published 25 articles, an h-index of 185, and a CiteScore of 10.9, evidencing its strong reputation in composite research. The growth observed in these journals suggests an increasing interest and recognition of the importance of LCF in sustainable material development. The publications in these journals contribute to advancing knowledge, driving innovation, and improving the technical properties and applications of these sustainable composites. It is the collective effort of these publications that has marked the last decade with a significant increase in the research and application of LCF-PHC as shown Table 3.

**Table 3:** Top ten Journals to publish for “lignocellulosic fibers; hybrid composites; polymeric composites”.

S. No	Journal published	TP (R%)	h-index	CiteScore 2021	SNIP	SJR	Publisher
1	Journal of Natural fibers	91 (8 %)	47	4.7	2.313	0.595	Taylor & Francis
2	Materials Today: Proceedings	60 (5,3 %)	69	3.2	0.774	0.445	Elsevier
3	Polymer Composites	57 (5 %)	94	6.7	1.146	0.672	Wiley-Blackwell
4	Polymers	43 (3,8 %)	113	7.2	1	0.72	MDPI
5	Materials Research Express	35 (3,1 %)	52	5.0	0.626	0.401	Institute of Physics Publishing
6	Composites Part B Engineering	32 (2,8 %)	184	23.2	2.675	2.3	Elsevier
7	Journal of Industrial Textiles	28 (2,5 %)	49	4.2	1.761	0.551	SAGE
8	Fibers and Polymers	27 (2,4 %)	65	3.9	0.823	0.451	Korean Fiber Society
9	Journal of Composite Materials	26 (2,3 %)	102	5.7	1.109	0.593	SAGE
10	Composite Structures	25 (2,2 %)	185	10.9	1.974	1.455	Elsevier

**TP:** Total journal publications; **R:** Rank; **SNIP:** Source normalized impact per paper; **SJR:** Journal Rank indicator.

**Fig. 9:**
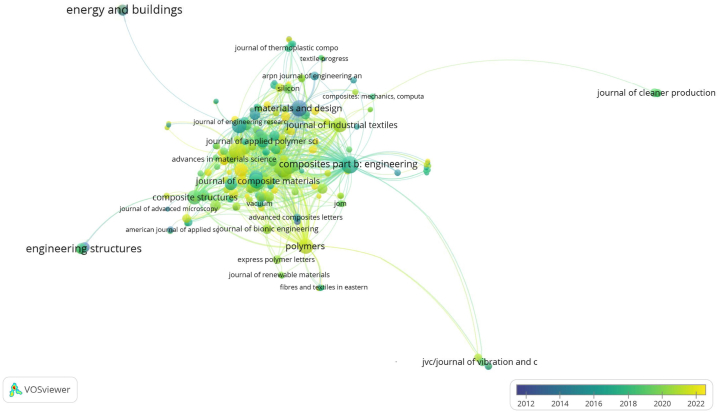
An overlay visualization of publications for journals (2012–2022) in lignocellulosic fiber-based polymeric and hybrid composites.

Fig. 9 shown an overlay visualization of publications from various journals related to LCF-PHC spanning from 2012 to 2022. In Fig. 9, the size of each node likely indicates the number of publications from that journal, while the color of each node represents the average publication year. Nodes colored closer to the blue end of the spectrum typically represent journals with earlier publications in the timeline (around 2012), and those closer to the yellow end represent journals with more recent publications (closer to 2022). Journals that are central and larger in the visualization are typically those with a higher volume of publications in this research area. These are likely to be the leading journals in the field of composite materials and engineering. The presence of Journal of Cleaner Production at the periphery with a color indicating more recent activity suggests an increasing interest in the sustainability aspects of lignocellulosic fiber composites in recent years.

### 5.6 Top cited authors

In the realm of lignocellulosic fiber-based polymeric and hybrid composites, the contributions of prominent authors have played a pivotal role in advancing research and knowledge. Over the decade from 2012 to 2022, several researchers emerged as leaders in this field, consistently enriching the body of literature as shown in Table 4.

**Table 4:** Top ten highly cited articles dealing with “lignocellulosic fibers; hybrid composites; polymeric composites” research from 2012 to 2022.

Article title	Author name	Journal published	Citations	Year
Mechanical property evaluation of sisal-jute-glass fiber reinforced polyester composites	Ramesh, M.; Palanikumar, K.; Reddy, K.	Composites Part B: Engineering	777	2013
Tensile and interfacial properties of unidirectional flax/glass fiber reinforced hybrid composites	Zhang, Yongli et al.	Composites Science and Technology	397	2013
Prediction of tensile properties of hybrid-lignocellulosic fiber composites	Venkateshwaran, N.; Elayaperumal, A.; Sathiya, G. K.	Composites Part B: Engineering	390	2012
Static and dynamic mechanical properties of alkali treated unidirectional continuous Palmyra Palm Leaf Stalk Fiber/jute fiber reinforced hybrid polyester composites	Shanmugam, D.; Thiruchitrambalam, M.	Materials & Design	322	2013
Evaluation of mechanical properties of abaca-jute-glass fibre reinforced epoxy composite	Ramnath, B. Vijaya et al.	Materials & Design	310	2013
Evaluation of mechanical and thermal properties of banana–flax based lignocellulosic fibre composite	Srinivasan, V. S. et al.	Materials & Design	267	2014
Hybrid lignocellulosic and glass fibers reinforced polymer composites material selection using Analytical Hierarchy Process for automotive brake lever design	Mansor, Muhd Ridzuan et al.	Materials & Design	246	2013
Fabrication and characterization of echinoidea spike particles and kenaf lignocellulosic fibre-reinforced Azadirachta-Indica blended epoxy multi-hybrid bio composite	V.R. Arun Prakash; Viswanthan, R.	Composites Part A: Applied Science and Manufacturing	168	2019
Environmental effects on the mechanical behaviour of pultruded jute/glass fibre-reinforced polyester hybrid composites	Akil, Hazizan Md et al.	Composites Science and Technology	220	2014
Manufacturing and characterization of sustainable hybrid composites using sisal and hemp fibres as reinforcement of poly (lactic acid) via injection molding	Pappu, Asokan; Pickering, Kim L.; Thakur, Vijay Kumar.	Industrial Crops and Products	202	2019

**Fig. 10:**
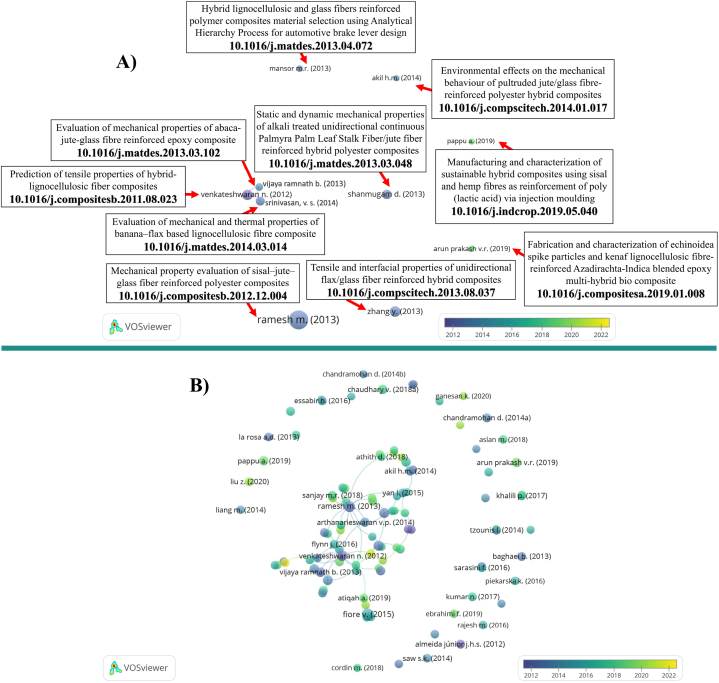
A) An overlay visualization of highly cited articles (2012–2022), and **B)** Visualization of top 100 cited articles in lignocellulosic fiber-based polymeric and hybrid composites.

Fig. 10A presents the top ten highly cited articles in the realm of LCF-PHC from 2012 to 2022, indicating the growing importance and impact of these works within the field. Ramesh, M., Palanikumar, K., and Reddy, K. R. lead the citation count with their article on the mechanical property evaluation of sisal-jute-glass fiber reinforced polymer composites, published in Composites Part B: Engineering with 777 citations. Their work contributes significantly to understanding the synergy of natural and synthetic fibers in composite materials [124]. Zhang, Yongli et al. have made a notable contribution with their research on the tensile and interfacial properties of unidirectional flax/glass fiber reinforced hybrid composites, which has been cited 397 times and also published in Composites Science and Technology. Their work enhances the understanding of fiber-matrix interactions [125] as shown in Fig. 10. Venkateswaran, N., Elayaperumal, A., Sathiya, G. K.’s study on prediction of tensile properties of hybrid lignocellulosic fiber composites, found in Composites Part B: Engineering, has gathered 390 citations, reflecting its role in predicting material behaviors in hybrid composites [126]. Shanmugam, D., Thiruchitrambalam, M.’s investigation into the static and dynamic mechanical properties of alkali treated unidirectional continuous Palmyra Palm Leaf Stalk fiber/polyester composites, cited 322 times and published in Materials & Design, contributes to the development of sustainable composite materials with enhanced mechanical properties as shown in Fig. 10 [127]. Ramnath, B. Vijaya et al.’s article on the evaluation of mechanical properties of abaca-jute-glass fiber reinforced epoxy composite in Materials & Design, with 310 citations, helps in understanding the performance of mixed natural and synthetic fiber composites [128]. Srinivasan, V. S. et al. have contributed with their study on the evaluation of mechanical and thermal properties of banana-flax based lignocellulosic fiber composite, also in Materials & Design, which has been cited 267 times as shown in Fig. 10. This work adds valuable insights into the thermal aspects of lignocellulosic fiber composites [129]. Mansor, Muhd Ridzuan et al.’s work on hybrid lignocellulosic and glass fibers reinforced polymer composites material selection using Analytical Hierarchy Process for automotive brake lever design, published in Materials & Design with 246 citations, integrates material selection processes with practical industrial applications [130]. V.R. Arun Prakash, Viswanthan, R. have made strides with their publication on the fabrication and characterization of kenaf LCF and echinoidea spike particles reinforced Azadirachta-Indica blended epoxy multi-hybrid composite in Composites Part A: Applied Science and Manufacturing, which has garnered 168 citations, pointing to the industry's interest in multi-hybrid composites [131]. Akil, Hazizan Md et al. have contributed to the field with their investigation of the environmental effects on the mechanical behavior of pultruded jute/glass fiber-reinforced polyester hybrid composites in Composites Science and Technology, receiving 220 citations, underlining the importance of environmental durability in composite performance [132]. Pappu, Asokan; Pickering, Kim L.; Thakur, Vijay Kumar.’s work on manufacturing and characterization of sustainable hybrid composites using sisal and hemp fibers as reinforcement of poly (lactic acid) via injection molding, cited 202 times and featured in Industrial Crops and Products, underscores the sustainable advances in composite manufacturing technologies [133]. The increasing citation counts for these articles reflect their foundational role and the ongoing relevance in the advancing field of LCF-PHC Fig. 10B. The researchers highlighted in this extensive overview have collectively made significant contributions to the field of composite materials, each addressing unique aspects of materials science, mechanical properties, and sustainability. Their collective contribution spans from the enhancement of mechanical properties to the application of sustainable materials in industry, underscoring a decade of significant progress and innovation from 2012 to 2022.

### 5.7 Timeline of research milestones

**Fig. 11:**
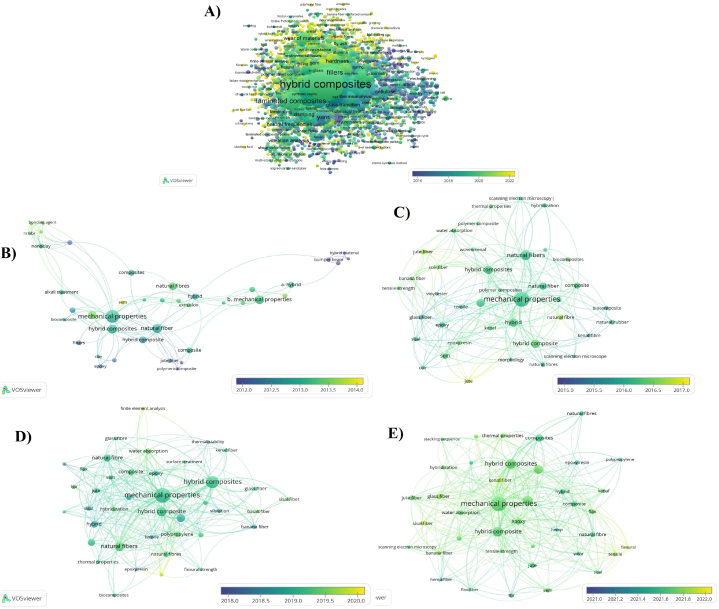
A) All keywords used in publications (2012–2022), and bibliometric analysis focusing on the co-occurrence of keywords within life cycle assessment research publications, accompanied by overlay visualization techniques. Time frames include: (B) 2012–2014; (C) 2015–2017; (D) 2018–2020; (E) 2021–2022."

In Fig. 11, was presented an overlay visualization generated using VOSviewer, illustrating the keyword occurrences within publications related to "natural AND fibers AND hybrid OR composites OR polymeric AND composites AND mechanical OR tensile, OR chemical, OR thermal OR morphological OR properties OR packaging, OR conventional, OR aerospace, OR automotive, OR sporting, OR marine OR oil AND industry, OR building, OR applications" from the years 2012–2022. This visualization offers valuable insights into the prevalent research themes, trends, and the evolution of these key concepts over the past decade. Fig. 11a exhibited the keywords used in publications, the overlay visualization highlights the frequency and co-occurrence patterns of keywords used in publications. Fig. 11B–E graphically depicts the networks formed by the clustering of keywords that frequently appear together. In order to accurately portray the central themes of the author keywords, specific thresholds were set according to how often each keyword appears. The size of the circles and the corresponding font sizes illustrate the prevalence of each keyword within the scholarly works, whereas the length of the connecting lines indicates the degree of association among the various subjects.

The research utilizes bibliometric visualization methods to map out the progression of research themes and key focus points within the domain of LCF-PHC over four separate time frames, covering the years 2012–2022. The initiation of each period marks a sequence of significant research achievements in the study of LCF-PHC. During each of the four-time spans, the fifteen most frequently cited author-keywords were accentuated. Table 5 presents a compilation of the top 15 author-keywords in the field of LCF-PHC from 2012 to 2022, showing the frequency of each keyword's occurrence in literature over time, indicating shifts in research focus and technological advancements in sustainable composites [134]. The thematic progression in the context of environmental impacts, end-of-life, life cycle assessment, and circular economy, using the frequency of keywords as indicators of evolving trends. The term Hybrid Composites has seen a substantial increase in occurrences, from 43 between 2012 and 2014 to 402 between 2021 and 2022 [[135], [136], [137]]. This indicates a significant growth in interest and research in combining LCF with other materials to enhance properties and sustainability. These composites are now critical in reducing environmental impacts by leveraging renewable resources and reducing reliance on non-renewable, petrochemical-based composites [[138], [139], [140], [141]].

**Table 5:** Top 15 author -keywords of the keyword (2012–2022) of lignocellulosic fiber-based polymeric and hybrid composites.

S. No	Keywords	Total Occurrences	2012–2014	2015–2017	2018–2020	2021–2022
1	Hybrid Composites	604	43	67	150	402
2	Natural Fibers	572	68	128	185	262
3	Reinforcement	544	41	96	207	280
4	Mechanical Properties	557	79	114	185	239
5	Tensile Strength	438	43	61	127	260
6	Fibers	355	67	124	75	118
7	Scanning Electron Microscopy	350	43	71	100	188
8	Fiber Reinforced Plastics	270	–	51	90	160
9	Composites	251	40	69	–	118
10	Reinforced Plastics	226	16	55	71	84
11	Hemp	211	17	40	56	98
12	Impact Strength	201	–	–	61	105
13	Water Absorption	190	–	–	58	88
14	Composite Materials	185	30	58	67	–
15	Jute Fibers	164	–	–	–	81

Occurrences of Natural Fibers have more than doubled from 68 between 2012 and 2014 to 262 between 2021 and 2022. This reflects a growing emphasis on using sustainable materials, as natural fibers are biodegradable and have a lower ecological footprint, which is essential for end-of-life considerations and life cycle assessments [142,143]. The consistent increase in these keywords (from 41 to 280 for Reinforcement and from 79 to 239 for Mechanical Properties) demonstrates the continuous improvement in processing and treatment of LCF to meet mechanical requirements [[144], [145], [146]]. These improvements help in creating composites that are not only environmentally friendly but also mechanically competitive, which is vital for their lifecycle performance and circular economy applications [[147], [148], [149], [150]]. The focus on Tensile Strength has increased significantly (from 43 to 260), showing an emphasis on enhancing the structural performance of composites [151,152]. Meanwhile, general research on Fibers has decreased (from 67 to 118), possibly indicating a maturation of foundational fiber research and a shift towards their applications [[153], [154], [155], [156]]. The increase in occurrences of scanning electron microscopy from 43 to 188 reflects the importance of advanced microscopy techniques in understanding the microstructure of composites [157,158]. This is crucial for predicting and assessing their environmental impacts and durability over their lifecycle [159].

The emergence of Impact Strength and Water Absorption in the literature indicates a nuanced exploration of the performance characteristics of composites in practical applications [160,161]. These properties are important for life cycle assessments, as they affect the longevity and environmental impact of composites [162,163]. The fluctuation in the keyword "composite materials" (from 30 to 0) suggests that the focus has shifted from generic composite materials to more specific types, such as those incorporating LCF [164]. These keywords collectively represent the core themes and areas of interest in the research related to LCF-PHC during the 2012–2022 period. The thematic progression in LCF-PHC shows a marked shift towards sustainability [[165], [166], [167]]. The research community's growing interest in hybrid composites, natural fibers, and reinforcement strategies reflects an integrated approach to develop materials that are not only high-performing but also environmentally responsible [168,169].

#### 5.7.1 The evolution of research themes

The timeline of research milestones and breakthroughs in lignocellulosic fiber composites during 2012–2022 was summarized.

• **2012–2014:** Early research primarily focused on characterizing LCF and exploring their compatibility with various polymer matrices. Increased interest in using LCF like jute, flax, and hemp as sustainable reinforcements in composites. Exploration of novel processing techniques for better fiber-matrix compatibility. Focus on improving the mechanical properties of lignocellulosic fiber composites for automotive applications. Advancements in surface modification techniques to enhance adhesion between LCF and polymer matrices. Research on the use of nanomaterials to further improve the properties of lignocellulosic fiber composites.
• **2015–2017:** Breakthroughs in manufacturing techniques led to the development of cost-effective and performance of lignocellulosic fiber composites. Growing research into hybrid composites combining LCF with synthetic or other lignocellulosic reinforcements. Increased understanding of the environmental benefits of lignocellulosic fiber composites, such as reduced carbon footprint.
• **2018–2020:** Numerous sectors are transitioning towards sustainable technology to enhance the equilibrium between environmental preservation and socioeconomic considerations, with notable successes in automotive, construction, and packaging sectors. Development of lignocellulosic fiber composites for non-conventional applications like 3D printing and aerospace components. Investigation into the use of waste or agricultural residues as potential sources of lignocellulosic fibers. Advancements in biodegradable polymer matrices for eco-friendly lignocellulosic fiber composites. Research on the fire resistance and flame-retardant properties of lignocellulosic fiber composites.
• **2021–2022:** Sustainable practices and circular economy principles gained prominence, influencing research to focus on end-of-life considerations and recycling of the lignocellulosic fiber composites. Adoption of artificial intelligence and machine learning for material design and optimization in lignocellulosic fiber composites. Continued exploration of circular economy principles, including recycling and upcycling, in composite materials. Increasing emphasis on the life cycle assessment and sustainability of lignocellulosic fiber composites in various industries.

The period between 2012 and 2022 has been transformative for lignocellulosic fiber composites, witnessing exponential growth, influential contributors, and a shift towards practical applications and sustainability [170]. This dynamic landscape sets the stage for further innovations and advancements in the coming years, as industries continue to embrace these eco-friendly materials.

##### 5.7.1.1 Red cluster: chemical and physical processing of lignocellulosic fibers

**Fig. 12:**
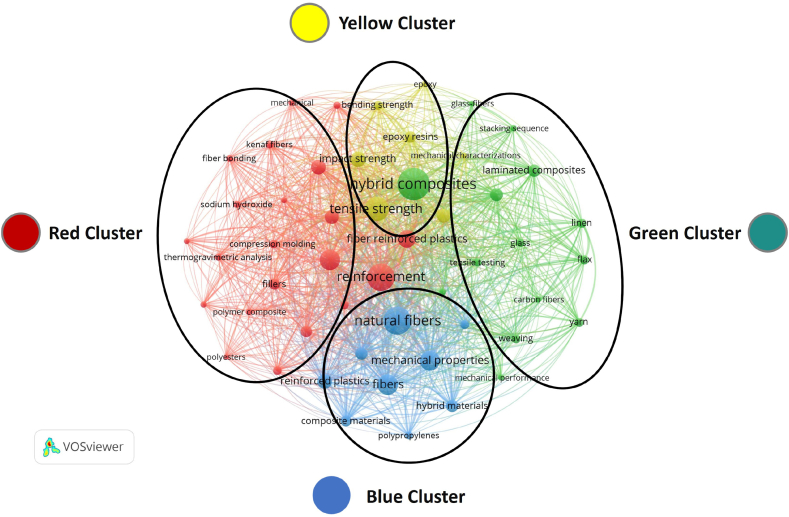
LCF-PHC keyword's theme clustering: 2012–2022.

This cluster showed to focus on the foundational aspects of polymer composites, particularly the role of LCF in enhancing the mechanical properties of these materials. Lignocellulosic fibers, which are derived from plant biomass, are a key area of research in the development of eco-friendly composite materials. Terms like kenaf fibers, fiber bonding, and sodium hydroxide indicate a focus on the chemical treatment and processing of natural fibers for integration into polymer matrices as showed in Fig. 12 thermogravimetric analysis suggests a concern with thermal stability, which is crucial for practical applications of these composites. Compression molding and fillers point towards manufacturing techniques and the incorporation of additives to improve performance as exhibited in Table 6.

**Table 6:** LCF-PHC keyword's theme clustering: 2012–2022.

Cluster	Main Keywords	Theme
Red	Kenaf fibers/fiber bonding/sodium hydroxide/compression molding/thermogravimetric analysis/polymer composite/polyesters	Chemical and physical processing of lignocellulosic fibers
Green	Hybrid composites/glass/carbon fibers/tensile strength/fiber reinforced plastics/reinforcement	Structural and mechanical aspects of hybrid composites
Blue	Mechanical properties/reinforced plastics fibers/composite materials/natural fibers/polypropylenes.	Mechanical properties and performance of lignocellulosic fiber-reinforced composites.
Yellow	Epoxy/glass fibers/impact strength	Traditional synthetic fiber-reinforced composites

##### 5.7.1.2 Green cluster: structural and mechanical aspects of hybrid composites

The central theme of this cluster is likely the development and characterization of hybrid composites, which are materials made by combining two or more different types of fibers or matrices to achieve desired properties. Hybrid composites and tensile strength are prominent, highlighting the focus on the mechanical performance of these materials as showed in Table 6. Fiber reinforced plastics and reinforcement suggest a strong interest in how LCF contribute to the strength of plastics as exhibited in Fig. 12. This cluster indicates a multidisciplinary approach, integrating knowledge from polymer science and material engineering to enhance composite materials' structural capabilities.

##### 5.7.1.3 Blue cluster: mechanical properties and performance of lignocellulosic fiber-reinforced composites

This cluster may center around the mechanical properties and performance evaluation of composite materials. Terms like mechanical properties, reinforced plastics fibers, and composite materials are indicative of studies on the structural and mechanical aspects of composites as showed in Fig. 12. Natural fibers show the importance of eco-friendly materials in this research area as exhibited in Table 6. The presence of polypropylenes, a type of plastic, suggests that this cluster is concerned with the combination of LCF with various polymer matrices to develop composites with superior properties.

##### 5.7.1.4 Yellow cluster: traditional synthetic fiber-reinforced composites

This is a smaller cluster, possibly focusing on specific types of fibers or treatments used in composites. Epoxy and glass fibers point towards research in traditional composite materials, which are reinforced with synthetic fibers as showed in Fig. 12. In the context of lignocellulosic fibers, this could imply research into the compatibility and comparative performance of natural fibers versus synthetic ones in polymer matrices as exhibited in Table 6 the map indicates a vibrant research field centered around the use of natural, LCF to enhance and innovate within the realm of polymer and hybrid composites. The clusters reflect a blend of interests ranging from the chemical treatment of natural fibers, their integration with polymers, the characterization of hybrid materials, and the evaluation of mechanical properties. The role of LCF in this context is critical, as they offer a renewable, less environmentally damaging alternative to synthetic fibers, while potentially providing comparable or superior material properties.

## 6 Composite manufacture

**Fig. 13:**
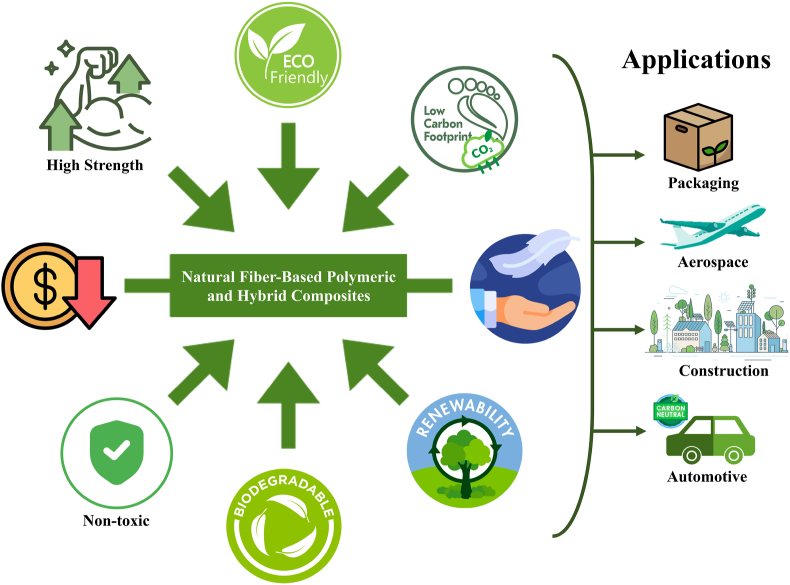
Applications and advantages of lignocellulosic fiber-based polymeric and hybrid composites.

In Fig. 13, lignocellulosic fiber composites have gained prominence in recent years due to their eco-friendly nature and impressive mechanical properties [[171], [172], [173], [174]]. These versatile materials find applications across various industries, offering sustainable and innovative solutions [[175], [176], [177], [178]]. Here, we explore the diverse applications of lignocellulosic fiber composites in automotive, construction, aerospace, and packaging as shown in Fig. 13 [[179], [180], [181]]. In the automotive industry lignocellulosic fiber composites are used to manufacture interior components such as door panels, dashboard trims, and seat backs [[182], [183], [184], [185]]. They provide a lightweight and aesthetically pleasing alternative to traditional plastics [[186], [187], [188], [189]]. The components of exterior Parts like bumpers, spoilers, and underbody panels benefit from lignocellulosic fiber reinforcements. These composites offer good impact resistance and reduce vehicle weight, contributing to fuel efficiency [[190], [191], [192]]. Some high-performance vehicles incorporate lignocellulosic fiber composites in structural elements, enhancing both strength and weight savings, as well as noise insulation lignocellulosic fiber composites are utilized in acoustic insulation materials, reducing noise levels inside the vehicle cabin [[193], [194], [195]]. In the construction industry, lignocellulosic fiber composites find application in the construction of eco-friendly building materials, including wall panels, flooring, and roofing [[196], [197], [198], [199]]. They offer insulation properties and contribute to energy efficiency. Sustainable furniture manufacturing often involves the use of lignocellulosic fiber composites for chair backs, table surfaces, and decorative elements, as well as infrastructure the composites play a role in strengthening and retrofitting concrete structures, extending their durability [200,201]. In the aerospace industry, the interior components of Lightweight lignocellulosic fiber composites are employed in aircraft interiors, reducing overall weight and enhancing fuel efficiency [202,203]. In non-structural aircraft parts, they are used in components like overhead bins, cabin partitions, and lavatory units, also some rotor blades in helicopters [204] and small aircraft incorporate LCF to reduce weight while maintaining strength [205,206]. While, in the packaging industry, lignocellulosic fiber composites are used to manufacture eco-friendly packaging materials, including boxes, trays, and pallets. They provide durability and sustainability. Some beverage container of companies utilizes lignocellulosic fiber composites in bottle caps and closures, reducing the environmental footprint as shown in Fig. 13. The applications of the lignocellulosic fiber composites extend beyond these industries, including sports equipment, marine, and consumer goods [207]. Their versatility, combined with their environmental benefits, makes them an attractive choice for a wide range of products and components as shown in Fig. 13. As research continues to advance, we can expect even more innovative applications of the lignocellulosic fiber composites in the future, contributing to sustainable and resilient industries. Lignocellulosic fiber composites have become increasingly popular in the automotive industry for several reasons as ability to reduce the overall weight of automotive components [[208], [209], [210]]. This, in turn, improves fuel efficiency and reduces emissions. Lignocellulosic fibers, such as jute, hemp, and flax, are renewable resources. Using them in composites aligns with the automotive industry's sustainability goals and reduces its carbon footprint as shown in Fig. 13 [211]. LCF can dampen noise and vibrations, contributing to quieter vehicle interiors and improved comfort due to enhanced acoustic properties [212,213]. Lignocellulosic fiber composites are biodegradable, making them environmentally friendly and easy to dispose of at the end of the vehicle's life cycle. In construction applications the lignocellulosic fiber composites offer several advantages as insulation because the composites made with LCF provide excellent thermal insulation properties, contributing to energy efficiency in buildings [[214], [215], [216]]. Still, the use of renewable LCF aligns with green building practices and sustainability standards, enhance the durability of construction materials, increasing their lifespan and reducing maintenance costs, and also aesthetic appeal they designed with various textures and colors, adding aesthetic value to construction projects [217,218]. In the aerospace sector, lignocellulosic fiber composites are gaining recognition for their potential benefits similar to the automotive industry, aerospace manufacturers appreciate the weight-saving properties of these composites, which contribute to fuel efficiency and payload capacity as shown in Fig. 13 [219]. LCF combined with advanced polymer matrices offer an excellent strength-to-weight ratio, making them suitable for aerospace components, and promoting the reduction of environmental impact aerospace companies are increasingly focused on sustainability, and the use of LCF aligns with eco-friendly initiatives, and cost efficiency because provide cost savings compared to traditional aerospace materials like carbon fiber [220,221]. There are no different packaging applications that have been found in the lignocellulosic fiber composites way into packaging materials for consumer and industrial applications adding biodegradation capacity to the product the packaging made from lignocellulosic fiber composites is biodegradable and compostable, reducing environmental impact and waste [[222], [223], [224]]. Another advantage of customization is that the composites are easily molded into various shapes and sizes, allowing for the customization of packaging designs, and companies enhance their sustainability image by using lignocellulosic fiber composites in packaging, appealing to eco-conscious consumers, and also protection, the composites offer good shock-absorbing properties, protecting fragile items during transportation [225,226]. In finally, lignocellulosic fiber composites have unique advantages in automotive, construction, aerospace, and packaging applications [[227], [228], [229]]. Their sustainability, weight reduction, insulating properties, and cost-effectiveness make them a promising choice for industries looking to reduce their environmental footprint while maintaining or improving performance as shown in Fig. 13 [230]. As research and development continue, was expect to see even more innovative uses of the lignocellulosic fiber composites in various sectors. In terms of emerging and innovative applications, the lignocellulosic fiber composites find applications in the manufacturing of sporting goods such as bicycles, skateboards, kayaks, canoes, and paddles. To incorporate lignocellulosic fiber composites combining durability with environmental sustainability, they offer a lightweight alternative to traditional materials. Also, have found applications in renewable energy that are explored for use in wind turbine blades (the limiting blade length is 26 m for lignocellulosic fiber composites [231], 45 m for glass fiber composites [232] and 107 m for carbon fibers composites [233]), due to their lightweight and durable properties. They contribute to the renewable energy sector's sustainability goals [234]. And, for use in consumer products utilized in the casings and housings of consumer electronics to reduce weight and environmental impact [235]. These emerging and innovative applications of the lignocellulosic fiber composites highlight their versatility and potential across various industries [[236], [237], [238]]. As sustainability and environmental concerns continue to grow, lignocellulosic fiber composites are likely to play an increasingly significant role in providing eco-friendly and high-performance solutions [239,240].

## 7 Performance

### 7.1 Fiber cost

Hybrid composites, incorporating both LCF and synthetic materials, have gained significant attention, due to their potential to address sustainability concerns and enhance material properties. In this discussion, was explored the trends in research related to hybrid composites incorporating lignocellulosic fibers, the commonly used secondary materials, and the performance enhancements achieved through hybridization [241,242]. One prominent trend is the increasing focus on sustainability and environmental friendliness. Researchers are incorporating lignocellulosic fibers, such as jute, flax, hemp, and sisal, to reduce the reliance on non-renewable resources and minimize the carbon footprint of composite materials [243]. Hybrid composites are being studied extensively to improve mechanical properties [244,245]. The combination of LCF with synthetic reinforcements, like glass or carbon fibers, is being explored to achieve a balance between lightweight and high-strength properties [[246], [247], [248]]. With growing awareness of the environmental impact of composites, research is also directed towards biodegradable matrices and lignocellulosic fiber composites that can be easily recycled or disposed of without causing harm to the environment obtaining biodegradability materials and end-of-life [249]. Researchers are exploring the multifunctional capabilities of hybrid composites. These materials can provide not only structural strength but also electrical conductivity, thermal insulation, and even self-healing properties, depending on the combination of fibers and matrices. Commonly used secondary materials in hybrid composites Thermosetting and thermoplastic polymer matrices, such as epoxy, polypropylene, and polyethylene, are commonly used in hybrid composites [[250], [251], [252]]. These matrices provide adhesion between LCF and other reinforcements. Glass fibers are frequently combined with LCF to create hybrid composites [[253], [254], [255], [256]]. They offer excellent mechanical properties and can enhance the overall strength and stiffness of the composite. For applications requiring high-performance composites, carbon fibers are often used in conjunction with lignocellulosic fibers. This combination provides a unique blend of lightweight and high-strength properties. Nanomaterials, such as nanoclays and carbon nanotubes, are incorporated into hybrid composites to further enhance mechanical, thermal, and electrical properties [[257], [258], [259], [260]]. These nanomaterials can be added to both the lignocellulosic fiber and synthetic components [[261], [262], [263]]. Performance enhancements achieved through hybridization promotes improvement mechanical properties, and the combination of LCF with synthetic reinforcements can lead to enhanced tensile strength, flexural strength, and impact resistance [[264], [265], [266]]. This is particularly valuable in applications where lightweight materials with high strength are required. Hybrid composites allow for weight reduction compared to using only synthetic materials. This is crucial in industries like automotive and aerospace, where fuel efficiency and performance are critical, also the LCF are often less expensive than synthetic counterparts as [267]. Incorporating them into composites can lead to cost savings while maintaining acceptable performance levels. By using lignocellulosic fibers, hybrid composites contribute to sustainability goals by reducing the reliance on non-renewable resources and lowering the environmental impact of the materials [268]. In the tailored properties the researchers customize hybrid composites to meet specific application requirements by adjusting the ratio and type of lignocellulosic fibers, secondary materials, and matrices. This flexibility is invaluable in various industries. The trends in research related to hybrid composites incorporating LCF reflect a growing emphasis on sustainability, improved mechanical properties, and multifunctionality [269]. The choice of secondary materials, such as polymer matrices, glass fibers, carbon fibers, and nanomaterials, plays a crucial role in achieving performance enhancements through hybridization [[270], [271], [272]]. As technology continues to advance, hybrid composites are likely to play a significant role in addressing the material needs of various industries while adhering to sustainability principles [273]. Lignocellulosic fiber hybrids (3.00–7.00 USD/kg), such as sisal-jute or flax-glass, are known for their sustainability and low cost [[274], [275], [276]]. They are often chosen for their environmentally friendly characteristics and affordability. However, their mechanical properties may not match those of synthetic counterparts. Glass fiber hybrid (4.00–10.00 USD/kg) are widely used in composite materials due to their moderate cost and excellent strength-to-weight ratio [277,278]. They offer good mechanical properties and are suitable for a range of applications, from automotive components to construction materials. Carbon fiber hybrids (10.00–25.00 USD/kg) are renowned for their exceptional strength and lightweight properties [279]. They are relatively expensive compared to other fibers but are favored in industries where high-performance composites are essential, such as aerospace and sports equipment. Aramid fiber hybrid (15.00–30.00 USD/kg), like Kevlar, are known for their outstanding strength and resistance to impact. They come at a higher cost but find applications in bulletproof vests, protective gear, and high-stress environments [280,281]. Basalt fiber hybrids (5.00–12.00 USD/kg) offer a balance between cost and performance [282]. They are valued for their resistance to high temperatures and fire. Applications include automotive parts and construction materials.

### 7.2 Matrix cost

Over the past decade, there has been a noticeable shift in the materials science landscape, with a growing emphasis on sustainable and eco-friendly solutions [283,284]. In this context, the integration of LCF into polymeric composites has gained significant traction, reflecting a broader trend toward environmentally conscious materials [283,285]. Table 7 provided lists five types of resins used in lignocellulosic fiber-based polymeric and hybrid composites. This article examines the evolving trends in research related to polymeric composites with LCF as reinforcement, shedding light on the types of polymers used and their influence on composite properties [[286], [287], [288]]. Lignocellulosic fibers, such as jute, flax, hemp, and sisal, offer a range of advantages, including renewability, biodegradability, and reduced environmental impact compared to traditional synthetic reinforcements like glass or carbon fibers [[289], [290], [291], [292]]. The Bio-based resins have a modulus range of 3.4 to 3.2 GPa and a relatively low cost of 2 USD to 5 USD per kg. With a density range of 1.0–1.50 g/cm³, they are not the stiffest materials available but are used in applications valuing sustainability, like packaging and consumer goods. Their growing use reflects a trend towards environmentally friendly materials. The thermoplastics resins are versatile with a modulus range of 0.3–3.5 GPa and cost between 1 USD to 3 USD per kg. They have a lower density (0.90–1.05 g/cm³) and are used across various industries, from automotive to electronics, indicating their adaptability and importance in manufacturing [293]. The polyurethane with a cost of 2 USD to 4 USD per kg and a density range of 1.05–1.25 g/cm³, polyurethane resins offer a modulus between 0.5 and 3 GPa. They are commonly used in construction, insulation, furniture, and automotive industries for their balance of flexibility and strength [294]. Positioned higher in terms of modulus (3.1–3.8 GPa) and cost (3 USD to 6 USD per kg), vinyl ester resins have a density of 1.2–1.4 g/cm³ and are used in marine and corrosion-resistant applications. They offer a good balance between cost and performance, particularly in harsh environments. At the top in terms of modulus (3.0–6.0 GPa) and cost (5 USD to 15 USD per kg), epoxy resins [295,296] have a density range of 1.1–1.6 g/cm³. They are used in high-performance applications such as aerospace and structural components due to their excellent strength (28–100 MPa) and adhesion properties [[297], [298], [299]]. Achieving competitive tensile strength, flexural strength, and impact resistance has been a primary focus [282,300]. The type of LCF and their chemical treatment can significantly impact the compatibility with these matrices and the overall performance of the composites. For instance, surface treatments on fibers can improve their adhesion to the resin matrix, enhancing the strength and durability of the final composite. This is particularly relevant for high-performance applications where the mechanical properties of the composite are critical. By selecting the appropriate resin type and treating LCF to optimize their interaction with the matrix, manufacturers can tailor the properties of the composites for specific applications. The choice of polymer matrix plays a crucial role in determining composite characteristics, and researchers continue to explore innovative combinations to meet the evolving demands of various industries [301,302]. As sustainability and eco-consciousness remain at the forefront, the future of polymeric composites reinforced with LCF holds promising potential for even more diverse and impactful applications.

**Table 7:** Estimated cost of matrices used to LCF-PHC in US Dollar (USD).

Resin type	Cost (USD/kg)^a^	Density (g/cm³)	Modulus (GPa)	Strength (MPa)	Applications	Ref.
Bio-based	2–5	1.0–1.50	3.4–3.2	35.6–49	Packaging, Consumer Goods	[303,304]
Thermoplastics	1–3	0.90–1.05	0.3–3.5	20–60	Automotive, Electronics	[305,306]
Polyurethane	2–4	1.05–1.25	0.5–3	40–50	Construction, Insulation, Furniture, automotive.	[305,307]
Vinyl Ester	3–6	1.2–1.4	3.1–3.8	69–86	Marine, Corrosion-resistant	[305,306]
Epoxy	5–15	1.1–1.6	3.0–6.0	28–100	Aerospace, Structural	[305,306]

a Cost data is highly variable in current market prices.

Selecting between synthetic and LCF is key in industries like textiles and packaging, driven by factors such as cost, ecological impact, and sustainability goals [308]. LCF are biodegradable, often more economical, and have established processing methods. They are renewable with a lower carbon footprint, aligning with sustainability targets like the UN's 2030 agenda for responsible production. Synthetic fibers, while not biodegradable and dependent on non-renewable resources, can benefit from scale economies but may have supply vulnerabilities and greater environmental impacts [309,310]. Balancing these aspects is crucial for making environmentally responsible choices in fiber use [[311], [312], [313], [314], [315], [316], [317], [318]]. The United Nations' 2030 agenda emphasizes SDGs, including responsible production and consumption (Goal 12) and climate action (Goal 13) [319,320].

### 7.3 Composite cost

Synthetic-based composites (4.00–10.00 USD/kg), such as polyester-fiber glass, are economical choices for many applications. They offer a good combination of mechanical properties and durability at a reasonable cost. Epoxy-based composites (6.00–15.00 USD/kg) are known for their excellent bonding strength and resistance to chemicals [321,322]. They are commonly used in aerospace, marine, and high-performance applications due to their moderate cost and versatility. Polyurethane-based composites (5.00–12.00 USD) offer flexibility, impact resistance, and good adhesion properties [[323], [324], [325]]. They are used in applications where toughness and durability are essential, such as automotive components and sports equipment. Thermoplastic-based composites (4.00–10.00 USD/kg), like polypropylene-glass, are cost-effective choices with the advantage of recyclability [326]. They find applications in automotive parts, consumer goods, and packaging. Also, the bio-based composites (7.00–18.00 USD/kg) are gaining popularity due to their eco-friendly nature as shown in Table 8 [327]. They are typically more expensive than traditional resins but offer sustainability benefits. These composites are used in environmentally conscious industries and products [[328], [329], [330]]. The choice of fiber and resin type for hybrid composites depends on the specific application's requirements, budget constraints, and sustainability goals. While some materials like carbon fiber and aramid come at a premium, they offer exceptional properties for high-performance applications [331]. Others like LCF and thermoplastics offer cost-effective and environmentally friendly options for a wide range of industries. Making the right selection involves considering factors such as mechanical performance, cost, environmental impact, and industry standards [332,333].

**Table 8:** Mechanical properties for some lignocellulosic fibers (2012–2022).

Lignocellulosic Fibers	Density (g/cm^3^)	Strain to failure (%)	Tensile Strength (MPa)	Young's Modulus (GPa)	Ref.
Cissus quadrangularis	1.38	7.52	146	1.58	[344]
Boehmeria Nivea	1.5	1.2–3.8	400–938	128	[338]
Cannabis sativa	1.45	1.3–4.7	580–1110	90	[339]
Vernonia elaeagnifolia	1.30	6.69	259.62	37.75	[345]
Elaeis guineensis	0.7–1.5	8–18	50–400	1–9	[201]
Musa textilis	1.5	10–12	980	72	[339]
Dichrostachys Cinerea	1.24	7–13	810–887	70.6	[58,59]
Epipremnum aurem	0.65	1.4–4.2	317–810	69.6	[54]
Acacia Leucophloea	1.39	1.38–4.24	317–1608	69.6	[60,61]
Trachelospermum jasminoides	1.4	1.0–3.4	197.2–675.2	55.3	[62]
Sida Cordifolia	1.33	2.7–3.1	680.2–727.7	44.9	[63]
Carica Papaya	0.78	1.2–1.6	5–541.1	44.4	[340,341]
Linum usitatissimum	1.50	2.7–3.2	345–1100	27.6	[338,346]
Corchorus olitorius	1.35	1.2–1.5	393–773	26.5	[338]
Thespesia Populnea	1.41	2.2–3.4	500.7–613.3	25.1	[69,342]
Agave sisalana	1.5	2–2.5	511–635	22	[343]
Fimbristylis globulosa	0.89	–	405–499	21.3	[71,72]
Conium maculatum	–	2.14–3.2	260.5–395.3	19	[79,80]
Red banana peduncle	0.90	1.56–1.64	426.6–453.4	17.4	[81]
Coccinia grandis	1.24	2.4–3	273	10.17	[74]
Hierochloe Odarata	1.16	1.4–3.4	70.3–141.1	3.6	[77]
Cereus Hildmannianus	1.36	0.44–2	2874.5–2920.5	3	[78]
Mucuna atropurpurea	1.08	2.21	274.6	2.88	[75]
Tridax procumbens	1.16	2.5–3.1	25.8	1	[76]

### 7.4 Mechanical properties of the LCF from 2012 to 2022

Table 8 exhibited a detailed list of mechanical properties for a range of LCF compared to traditional synthetic fibers like Aramid, S-glass, E-glass, and Carbon fibers. LCF are gaining popularity as sustainable alternatives in composite materials, and their mechanical properties are crucial for their selection in various applications. Carbon fibers with the highest tensile strength (4000 MPa) and modulus (240 GPa) listed, carbon fibers are the benchmark for high-performance applications but lack the sustainability of lignocellulosic options [334,335]. Considering the structural rigidity, linked to the material's modulus of elasticity, predominate in the design of composites, suggesting an unlikely replacement of natural fibers by carbon fiber. Cissus quadrangularis fibers shows a wide range of tensile strengths (1857–5330 MPa) comparable to synthetic fibers and could be used in applications where stiffness and strength are needed, potentially as a sustainable alternative to E-glass [336,337]. Boehmeria nivea fiber has a lower modulus (128 GPa), it can be a viable alternative for less critical applications where a moderate modulus is sufficient, unlike the high-performance applications of Aramid fibers [338]. Cannabis sativa fibers, known for its sustainability, offers a good tensile strength (580–1110 MPa) that could serve as an alternative to S-glass in certain contexts, especially given its lower environmental footprint [339]. Musa textiles commonly known as Manila hemp, it has a high tensile strength (980 MPa) and could be used in composites where S-glass is typically used, though it has a lower modulus (72 GPa) [339]. Dichrostachys cinerea fibers with a modulus (70.6 GPa) close to that of E-glass, it could be considered for certain applications that require moderate stiffness and strength [58,59]. The E-glass fibers are a common synthetic choice with a high modulus (70 GPa) but can be substituted with certain LCF that offer a more sustainable profile without significantly compromising on mechanical properties [307]. Epipremnum aureum fibers have lower modulus fiber may be more suitable for non-structural applications where E-glass might be overqualified [54]. Acacia leucophloea fibers and Aramid fibers both have high strength and modulus but Aramid fibers are synthetic and non-renewable, unlike the natural and more sustainable Acacia fibers [60,61,307]. Trachelspermum jasminoides fibers and Sida cordifolia fibers could serve as sustainable substitutes in some applications traditionally served by S-glass and other synthetic fibers due to their reasonable mechanical properties [62,63]. Carica papaya fibers have lower mechanical properties suggest they could be used for lightweight applications where natural fiber composites are favored over more robust E-glass or S-glass composites [340,341]. Thespesia populnea fibers its tensile strength suggests it could be a sustainable alternative in certain medium-load applications where S-glass is used [69,342]. Agave sisalana fibers, Fimbristylis globulosa fibers, and Conium maculatum [79,80] fibers have lower modulus values and be alternatives in less demanding applications compared to high-modulus synthetic fibers like Aramid [71,72,343].The red banana fibers compared to E-glass fibers has a lower modulus but could still be used in place of E-glass for specific applications requiring sustainability over ultimate performance [81,307]. Hierochoe odorata fibers due to their flexibility, these could be used in niche applications where synthetic fibers would be less ideal due to environmental considerations [77]. Mucuna atropurpurea fibers offer high tensile strengths which could make them potential sustainable alternatives to certain synthetic fibers in specific applications [75]. Tridax procumbens fibers with the lowest modulus and strength, it is more likely to be suitable for low-load carrying applications compared to synthetic fibers [76]. Cissus quadrangularis, with a density of 1.38 g/cm³ and strength of 146 MPa with 7.52 % elongation and 1.58 GPa Young's modulus, offers a moderate density, suitable for situations where lightness is crucial. Although its tensile strength is relatively low, its satisfactory failure tolerance and Young's modulus make it suitable for applications that demand stiffness, but not critically. On the other hand, Cereus hildmanianus exhibits a slightly lower density of 1.36 g/cm³, but an exceptional tensile strength ranging from 2874.5 to 2920.5 MPa with 0.44–2 % elongation and a Young's modulus of 3 GPa. Its main advantage lies in the high tensile strength and the ability to withstand high stresses until failure, making it ideal for applications where strength is fundamental, such as structural components. Furthermore, its significantly higher Young's modulus indicates greater stiffness, making it suitable for applications requiring dimensional stability and resistance to deformation. LCF offer a compelling sustainable alternative to synthetic fibers like Aramid, S-glass, and E-glass, particularly in applications where environmental impact is a significant concern. Their use is informed by a trade-off between mechanical properties and ecological benefits. As the push for sustainable materials grows, LCF are becoming more prominent in the material selection process for composite manufacturing, with ongoing research improving their properties and expanding their application range. When enhanced for compatibility with different matrices, they offer eco-friendly alternatives with desirable mechanical properties for a broad range of industrial applications. The values cited are crucial for material scientists and engineers when selecting fibers for specific composite applications, ensuring the materials meet the required performance standards while aligning with sustainability objectives.

## 8 Sustainable composites

### 8.1 Environmental impacts

Over the decade, there has been a marked shift towards minimizing the environmental footprint of composite materials. Natural fibers have been increasingly incorporated into composites due to their lower greenhouse gas emissions during production, renewable nature, and biodegradability [347]. The technical properties of both lignocellulosic and synthetic fibers have been a focal point for composite material innovation. While LCF like hemp, flax, and jute have seen increased use due to their sustainability benefits, their lower tensile strength and modulus have historically limited their applications. However, advancements in treatment processes and hybrid composites, which combine LCF with synthetic ones or use them with advanced matrix systems, have improved their functional properties [348]. The ecological impact of synthetic fibers, while more significant due to energy-intensive production and reliance on petrochemicals, has been mitigated somewhat by advances in cleaner production technologies and increased energy efficiency.

### 8.2 End-of-Life

End-of-life (EOL) considerations for composites have become a central issue in sustainable materials engineering. Synthetic fibers such as carbon, glass, and aramid continue to dominate sectors where high strength and modulus are critical, like aerospace and automotive [349]. Carbon fiber technology, in particular, saw significant advancements in production processes, leading to a reduction in costs and an expansion in its use beyond high-end applications. The ecological impact of fiber production has become increasingly important in the last decade, with a stronger emphasis on manufacturing processes and life cycle assessments. LCF have been championed for their lower environmental impact, being renewable and biodegradable. Their integration into bio-based composites has been an area of substantial growth, contributing to the development of more sustainable materials [350]. For synthetic fibers, the period saw a push towards reducing the carbon footprint of their production processes and improving recycling technologies. For example, carbon fiber recycling became more commercially viable, and processes for recycling glass and aramid fibers improved, though challenges remain. Natural fiber composites offer the advantage of biodegradability, potentially reducing landfill waste. In contrast, the EOL of synthetic fiber composites has seen innovation in recycling processes, particularly for carbon fiber, where recycled fibers retain substantial structural properties and can be reused in new composites [351].

### 8.3 Life cycle assessment (LCA)

Life Cycle Assessments have grown in importance and complexity, evaluating the environmental impacts associated with all the stages of a product's life from cradle to grave [352]. Natural fiber composites often show a more favorable LCA due to lower resource extraction impacts and end-of-life options [353]. However, the high performance of synthetic fiber composites can lead to reduced environmental impacts over the product's use phase, particularly in applications like automotive and aerospace, where weight reduction translates into energy savings. Natural fibers became more cost-effective, not only due to their lower material costs but also due to a greater focus on local sourcing and production, which aligns with sustainability and ESG goals [354]. The economic benefits were further enhanced by integrating these fibers into new composite applications, such as non-structural automotive parts and packaging. Synthetic fibers' costs were influenced by fluctuating petroleum prices, innovation in production technologies, and economies of scale. Despite higher costs, the performance benefits they provide, particularly in carbon fiber, justified their selection in many advanced engineering applications [355].

### 8.4 Circular economy

The concept of a circular economy has been increasingly applied to composite materials, with a focus on designing products for a lifecycle of reuse, remanufacturing, or recycling [356]. The 2012–2022 period has seen advancements in the recyclability of synthetic fibers and the development of bio-based resins that complement natural fibers in composites, supporting the creation of materials that fit into a circular economy model [357]. The availability of LCF improved due to increased cultivation of fiber crops and utilization of agricultural waste. This made them a more reliable resource for composite manufacturers looking for sustainable material sources. Synthetic fibers, particularly carbon fiber, saw an increase in production capacity, with new facilities coming online and existing ones expanding to meet growing demand [358]. The decade also witnessed the development of alternative raw material sources for synthetic fibers, such as recycling and bio-based precursors. The period from 2012 to 2022 has witnessed significant progress in sustainable composites. This progress has been driven by advancements in fiber treatments, manufacturing processes, recycling technologies, and a comprehensive understanding of environmental impacts through LCA. The industry's movement towards a circular economy has been underpinned by both incremental improvements and disruptive innovations, which have broadened the scope and improved the sustainability of composite materials [359].

### 8.5 Balancing technical requirements, economic considerations, processability, availability, and ecological impacts

**Fig. 14:**
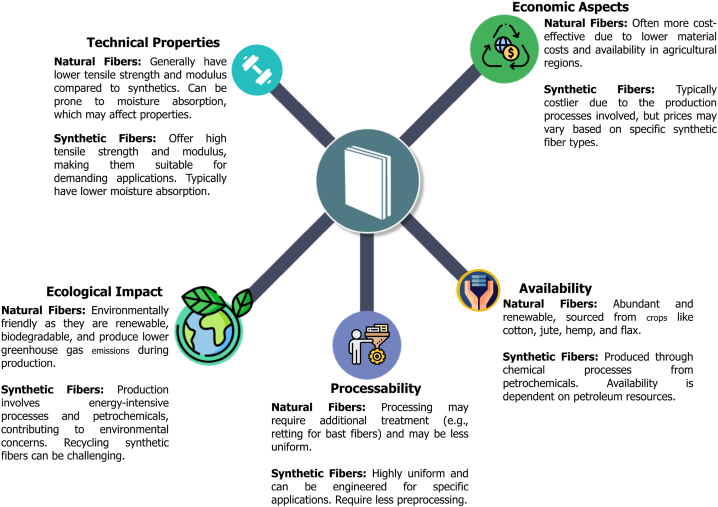
Comparison of technical, economic, processability, availability, and ecological characteristics between lignocellulosic fibers vs. synthetic fibers.

The choice between lignocellulosic and synthetic fibers should be a strategic one, balancing technical requirements, economic considerations, processability, availability, and ecological impacts as shown in Fig. 14 [23]. For example, LCF may be preferable for textiles where comfort and biodegradability are critical, while synthetic fibers may be more suitable for industrial applications requiring strength and durability [360]. In conclusion, the choice between lignocellulosic and synthetic fibers is multifaceted and depends on various factors. It's essential to consider the intended application and balance technical, economic, processability, availability, and ecological characteristics as exhibited in Fig. 14. Additionally, aligning with global sustainability initiatives like the 2030 agenda is increasingly important, as responsible production and consumption practices are essential for a more sustainable future. Fig. 14 shown a summary of the characteristics of lignocellulosic and synthetic fibers, which are crucial components in the development of polymer and hybrid composites. To generate a well-founded text focusing on the evolution and impact of these fibers in polymer and hybrid composites from 2012 to 2022, we'll examine each aspect presented in Fig. 14 in the context of developments over that decade. Between 2012 and 2022, the field of PHC witnessed significant advancements. The drive for sustainability led to increased utilization and enhancement of natural fibers, while synthetic fibers continued to evolve, offering unmatched technical properties for high-performance applications. The development of new matrix systems and treatment processes facilitated the creation of composites that were not only stronger and lighter but also more environmentally friendly. The progress in this decade laid a foundation for the next generation of composites, balancing performance with ecological and economic considerations [361].

## 9 Applications

Lignocellulosic fiber-based polymer and hybrid composites are garnering attention for their eco-friendly properties across various industries, yet they must surmount specific challenges to expand their application spectrum. Inherent susceptibilities, such as moisture absorption affecting stability, and limited durability against UV rays, microbes, and chemicals, constrain their use [362]. The inconsistency in fiber quality and the energy demands of processing these natural fibers further complicate their widespread adoption, alongside their diminished heat resistance. Advancements in enhancing these composites' moisture resistance via innovative surface treatments and chemical modifications are imperative [363]. Research should also delve into lignocellulosic enhancers that increase their environmental tenacity. Agricultural and genetic refinements could standardize fiber quality, while efficient processing techniques could escalate their market viability. Hybrid composites that meld lignocellulosic with synthetic fibers could harness the advantages of both. In packaging, the drive is towards diminishing plastic pollution through lignocellulosic composites. The automotive industry is integrating them into non-structural components, not only for their lightweight nature aiding fuel efficiency but also for improving vehicle comfort by reducing noise, vibration, and harshness (NVH) [364]. The construction sector is adopting them for insulation, leveraging their environmental benefits. Aerospace applications are emerging, although the specifics of their use in this sector are still being explored due to the stringent performance requirements. Innovation is key, with bio-based additives enhancing compliance with standards and digital manufacturing methods offering customized, industry-specific solutions. The path forward includes establishing effective recycling processes and thoroughly evaluating the lifecycle of these composites to solidify their ecological edge. To facilitate their broader use, addressing these challenges and driving innovation are pivotal. As research progresses, lignocellulosic fiber composites are poised to make substantial contributions towards a sustainable future in diverse fields such as packaging, construction, renewable energy, automotive, and aerospace [365,366].

## 10 Findings

• **Research Growth:** There has been a significant increase in research activity related to LCF-PHC from 2012 to 2022, with a marked rise in publications and citations, reflecting the growing importance and recognition of this field.
• **Key Materials:** The most commonly studied lignocellulosic fibers include jute, hemp, flax, and sisal, which have been widely examined for their reinforcement capabilities in polymeric composites.
• **Industry Applications:** LCF-PHC has found applications across various industries, including automotive, aerospace, construction, and packaging. These composites are valued for their mechanical properties, lightweight, and environmental benefits.
• **Influential Authors and Institutions:** The analysis identifies key authors and institutions contributing to the field, with significant research outputs from countries like India, Malaysia, China, and Brazil.
• **Sustainability and Environmental Impact:** The adoption of LCF in composites significantly reduces the environmental impact compared to traditional synthetic fibers, promoting a circular economy approach and supporting socio-economic development in rural areas.
• **Technological Advancements:** Advances in fiber treatment methods, such as alkaline, silane, acetylation, and enzymatic treatments, have improved fiber-matrix adhesion and overall composite performance.

## 11 Conclusions and future outlook

This decade-long bibliometric review, spanning from 2012 to 2022, has provided a thorough exploration into the utilization of LCF-PHC. The emerging topic clusters reveal the cross-disciplinary scope of research in lignocellulosic fibers, and polymeric and hybrid composites. An upswing in publications related to LCF-PHC suggests potential issues with research quality, yet the increase in sponsored research articles demonstrates a complex and evolving field, shaped by factors like SDGs, economic considerations, and ecological consciousness. The deepening cooperation among scholars, industry leaders, and governmental bodies has led to a surge in financial support. This concerted effort in research communities heralds ongoing expansion in the field of lignocellulosic fiber-based polymeric and hybrid composites. Over the past decade, India has emerged as a front-runner in this domain, surpassing the efforts of Malaysia, China, Brazil, Saudi Arabia, and the United States, thanks to its broad spectrum of institutions and academic partnerships. The international network of coauthors spans academic and research entities, with notable contributions from the developing world. The terrain of scientific journals has also shifted, with new publications gaining prominence and signaling a transformation in the landscape of LCF-PHC research throughout the last ten years. However, the findings of this analysis offer an expansive view of the development of LCF-PHC over four distinct intervals within the years 2012–2022. The bibliometric review provided by this study illuminates the robustness, evolution, and emerging patterns within this research area. It highlights the cross-disciplinary reach of lignocellulosic fiber-based polymeric and hybrid composite studies, which have been propelled by international cooperation and an adaptive publication landscape. This investigation presents key observations on the direction and momentum of LCF-PHC research over the past ten years, thereby enriching the progression of this scientific domain. Future research should focus on addressing the remaining challenges in processing techniques, improving fiber-matrix compatibility, and expanding applications in emerging fields such as 3D printing and aerospace. The continuous evolution in this domain underscores the critical role of LCF-PHC in advancing sustainable materials science and engineering.

## Ethical approval

Not Applicable.

## Funding

Not Applicable.

## Data availability statement

Data included in article/supp. material/referenced in article.

## CRediT authorship contribution statement

**Caroliny M. Santos:** Writing – review & editing, Writing – original draft, Visualization, Validation, Software, Resources, Methodology, Investigation, Funding acquisition, Formal analysis, Data curation, Conceptualization. **Thiago F. Santos:** Writing – review & editing, Writing – original draft, Visualization, Validation, Software, Resources, Methodology, Investigation, Funding acquisition, Formal analysis, Data curation, Conceptualization. **H Jeevan Rao:** Writing – review & editing, Visualization, Methodology, Data curation, Conceptualization. **F. Higor V.A. Silva:** Writing – review & editing, Visualization, Validation, Methodology, Investigation, Funding acquisition, Formal analysis, Data curation, Conceptualization. **Sanjay Mavinkere Rangappa:** Writing – review & editing, Visualization, Validation, Methodology, Investigation, Formal analysis, Data curation, Conceptualization. **Pawinee Boonyasopon:** Writing – review & editing, Visualization, Validation, Software, Methodology, Investigation, Funding acquisition, Formal analysis, Data curation, Conceptualization. **Suchart Siengchin:** Writing – review & editing, Validation, Supervision, Methodology, Formal analysis, Conceptualization. **D.F.S. Souza:** Writing – review & editing, Writing – original draft, Visualization, Validation, Methodology, Investigation, Formal analysis, Data curation, Conceptualization. **J.H.O. Nascimento:** Writing – review & editing, Visualization, Validation, Supervision, Software, Resources, Project administration, Methodology, Investigation, Funding acquisition, Formal analysis, Data curation, Conceptualization.

## Declaration of competing interest

The authors declare the following financial interests/personal relationships which may be considered as potential competing interests:The authors declare the following financial interests/personal relationships which may be considered as potential competing interests:The Corresponding Author of this paper, Sanjay Mavinkere Rangappa, works as an Associate Editor at Heliyon Materials Science.
